# Seasonal nitrogen remobilization and the role of auxin transport in poplar trees

**DOI:** 10.1093/jxb/eraa130

**Published:** 2020-03-12

**Authors:** Gen Li, Rongshoung Lin, Chioma Egekwu, Joshua Blakeslee, Jinshan Lin, Emily Pettengill, Angus S Murphy, Wendy A Peer, Nazrul Islam, Benjamin A Babst, Fei Gao, Sergey Komarov, Yuan-Chuan Tai, Gary D Coleman

**Affiliations:** 1 Department of Plant Science and Landscape Architecture, University of Maryland, College Park, USA; 2 OARDC Metabolite Analysis Center, Department of Horticulture and Crop Science, The Ohio State University, Wooster, USA; 3 Department of Environmental Science and Technology, University of Maryland, College Park, USA; 4 College of Forestry, Agriculture and Natural Resources, University of Arkansas at Monticello, Monticello, USA; 5 Department of Radiology, Washington University in St. Louis, St. Louis, USA; 7 Michigan State University, USA

**Keywords:** Auxin, bark storage proteins, nitrogen remobilization, *Populus*, seasonal nitrogen cycling

## Abstract

Seasonal nitrogen (N) cycling in *Populus,* involves bark storage proteins (BSPs) that accumulate in bark phloem parenchyma in the autumn and decline when shoot growth resumes in the spring. Little is known about the contribution of BSPs to growth or the signals regulating N remobilization from BSPs. Knockdown of BSP accumulation via RNAi and N sink manipulations were used to understand how BSP storage influences shoot growth. Reduced accumulation of BSPs delayed bud break and reduced shoot growth following dormancy. Further, ^13^N tracer studies also showed that BSP accumulation is an important factor in N partitioning from senescing leaves to bark. Thus, BSP accumulation has a role in N remobilization during N partitioning both from senescing leaves to bark and from bark to expanding shoots once growth commences following dormancy. The bark transcriptome during BSP catabolism and N remobilization was enriched in genes associated with auxin transport and signaling, and manipulation of the source of auxin or auxin transport revealed a role for auxin in regulating BSP catabolism and N remobilization. Therefore, N remobilization appears to be regulated by auxin produced in expanding buds and shoots that is transported to bark where it regulates protease gene expression and BSP catabolism.

## Teaser

This study establishes a role for poplar bark storage protein catabolism and N remobilization to shoot growth following dormancy, and a regulatory role for auxin in seasonal N remobilization.

## Introduction

Nitrogen (N) is a major nutrient that impacts plant growth and productivity. Forest trees and other perennial plants acquire, assimilate, and redistribute N during growth but have the additional feature of seasonal N redistribution where N is mobilized from senescing leaves to perennating storage tissues and N is remobilized from storage tissues when growth resumes (Staswick, 1994; Cooke and Weih, 2005; Babst and Coleman, 2018). Because seasonal N cycling occurs annually in deciduous forest trees, N is retained and reused for longer periods of time (Millard *et al.*, 2006; Millard and Grelet, 2010) which contributes to the economics of N use and provides a competitive advantage in N-limited conditions (Chapin *et al.*, 1990; Millard and Grelet, 2010). Consequently, seasonal N cycling is a major factor for N-use efficiency (NUE) (Vitousek, 1982; Chapin and Kedrowski, 1983; Aerts, 1990; Cánovas *et al.*, 2018), and is an important trait in the development of perennial lignocellulosic energy feedstocks (Karp and Shield, 2008; Allwright and Taylor, 2016). Despite the importance of seasonal N cycling to N allocation and partitioning, the contribution of stored N to growth and the mechanisms involved in source to sink remobilization of stored N remain poorly understood in trees.

Seasonal N cycling in the deciduous temperate tree poplar (*Populus*) involves the accumulation of 32 kDa bark storage proteins (BSPs) in protein storage vacuoles of bark phloem parenchyma and xylem ray cells during autumn and the disappearance of these proteins when shoot growth commences in the spring. Poplar BSPs are encoded by three genes (*BSPA*, *BSPB*, and *BSPC*) that belong to a family of nucleoside phosphorylase-like proteins of a larger 13 member gene family (Pettengill *et al.*, 2013). *BSP* expression and protein accumulation are regulated by environmental factors including short-day (SD) photoperiods, N availability, and low temperature (Coleman et al., 1991, 1992, 1994; Zhu and Coleman, 2001*a*, b; Wildhagen *et al.*, 2010; Pettengill *et al.*, 2013). Two phases of N remobilization occur during poplar seasonal N cycling. During autumn leaf senescence, leaves transition to N sources with N transported from leaves to bark phloem parenchyma and xylem ray cells which serve as N sinks (Sauter *et al.*, 1988; Wetzel *et al.*, 1989b). Once growth commences in the spring, bark phloem parenchyma and xylem rays become N sources where N from catabolized BSPs is remobilized to growing sink tissues (Wetzel *et al.*, 1989a; Coleman *et al.*, 1993).

Glutamine (Gln) has an important role in N source/sink transitions in poplar. Gln accumulates in poplar leaves during senescence and is the major amino acid transported during autumn N remobilization (Hörtensteiner and Feller, 2002; Couturier *et al.*, 2010). Gln is also the most abundant amino acid found in xylem during N remobilization from bark to growing shoots (Sauter and van Cleve, 1992). The cationic amino acid transporter CAT11 appears to be involved in phloem loading of Gln in senescing leaves (Couturier *et al.*, 2010) and may also have a role in unloading Gln to bark phloem parenchyma (Babst and Coleman, 2018). Besides it role in N transport, Gln also appears to have a role in N signaling during seasonal N cycling (Zhu and Coleman, 2001*a*).

Poplar BSP catabolism and N remobilization require communication between the N sink established by expanding buds and shoots with bark storage tissues (Coleman *et al.*, 1993), and is accompanied by extensive changes in the bark proteome including increased abundance of papain-like cysteine proteases, serine carboxypeptidases, and aspartyl proteases (Islam *et al.*, 2015). However, the nature of the signal between expanding buds and shoots and bark storage tissues has yet to be discovered. Here, transgenic and physiological approaches were used to alter BSP accumulation and shoot sink competition to examine the role of BSP storage and N remobilization to shoot growth. The role of long-distance transport of auxin from expanding buds and shoots to bark was found to play a significant role in BSP catabolism and N remobilization. Furthermore, transported auxin appears to be involved in mediating the expression of specific protease genes that could be involved in BSP catabolism.

## Materials and methods

### Plant material and experimental manipulation of N remobilization

*Populus trichocarpa* (Nisqually) and *Populus tremula*×*P. alba* hybrid clone INRA 717-1B4 were used in this study. BSP accumulation, catabolism, and N remobilization were experimentally manipulated using controlled-environmental conditions as previously described (Islam *et al.*, 2015). Briefly, plants are treated with SDs (8 h light/16 h dark) for 8 weeks at 20 °C to induce BSP accumulation, growth cessation, and bud dormancy. Following the SD treatment, an additional 5 weeks of SDs with the temperature reduced to 10 °C day/4 °C night (SD-LT) induces leaf senescence and abscission. An additional 7 weeks in the dark at 4 °C was used to overcome dormancy. Renewed shoot growth, BSP catabolism, and N remobilization were then initiated by transferring plants to long days (LDs; 16 h light/8 h dark) at 20 °C.

### N availability growth experiments

Rooted *in vitro* propagated plants of *P. tremula*×*P. alba* clone INRA 717 IB4 were transferred to a greenhouse and grown in 6.0 liter pots containing soilless medium (Sunshine LC1) under LD conditions (16 h light, 8 h dark) at 20 °C for 10 weeks and fertilized weekly with 500 ml of nutrient solution (8.3 mM KCl, 8.3 mM CaCl_2_·2H_2_O, 1 mM MgSO_4_·7H_2_O, 0.5 mM KH_2_PO_4_, 44.8 μM EDTA Fe (III) [C_10_H_12_FeN_2_NAO_8_], 23.1 μM H_3_BO_3_, 4.5 μM MnCl_2_·4H_2_O, 4 μM ZnCl_2_, 2.7 μM CuCl_2_·2H_2_O, 52 nM Na_2_MbO_4_·2H_2_O) containing 1.56 mM NH_4_NO_3_. Supplemental lighting to extend photoperiods to an LD (minimum of 16 h of light) was provided by high-pressure sodium lamps. After 10 weeks, uniform plants were randomly assigned to one of five N treatments and fertilized three times per week with 500 ml of a nutrient solution containing either 1.56, 3.12, 6.25, 12.5, or 25 mM NH_4_NO_3_ with three replicates per treatment. Ten weeks after N treatment, plants were destructively harvested and shoot growth parameters were measured.

### Shoot competition experiments during N remobilization

*Populus trichocarpa* plants were established from softwood cuttings, and rooted plants were transferred to 6.0 liter pots containing soilless medium (Sunshine LC1). Plants were grown in an LD (16 h light, 8 h dark) greenhouse for 6 weeks and fertilized at the beginning of LD growth with 2 g of 30 d controlled-release fertilizer (18-3-3, Nutricote). BSP accumulation, growth cessation, bud dormancy, and dormancy release were as described above except that the 4 °C treatment in the dark to overcome dormancy was extended to 10 weeks. Following low temperature dormancy release, plants of uniform height were selected and the apical bud was removed. Plants were then randomly assigned to one of the treatments used to manipulate sink competition. Treatments consisted of (i) plants with only one, three, six, nine, or 12 axillary buds; (ii) plants with all axillary buds removed but retaining one, five, or 10 sylleptic shoots; and (iii) plants with one or 10 axillary buds combined with either one or 10 sylleptic shoots. After bud/shoot removal, plants were placed in an LD greenhouse and, after 5 weeks, leaf number, leaf area, stem length, number of new sylleptic shoots, and length of new sylleptic shoots were measured for shoots arising from axillary buds, while leaf area, number of new sylleptic shoots, and length of new sylleptic shoots were determined for growth arising for previously formed sylleptic shoots.

### Generation and analysis of BSP RNAi poplars

**Fig. 1.**
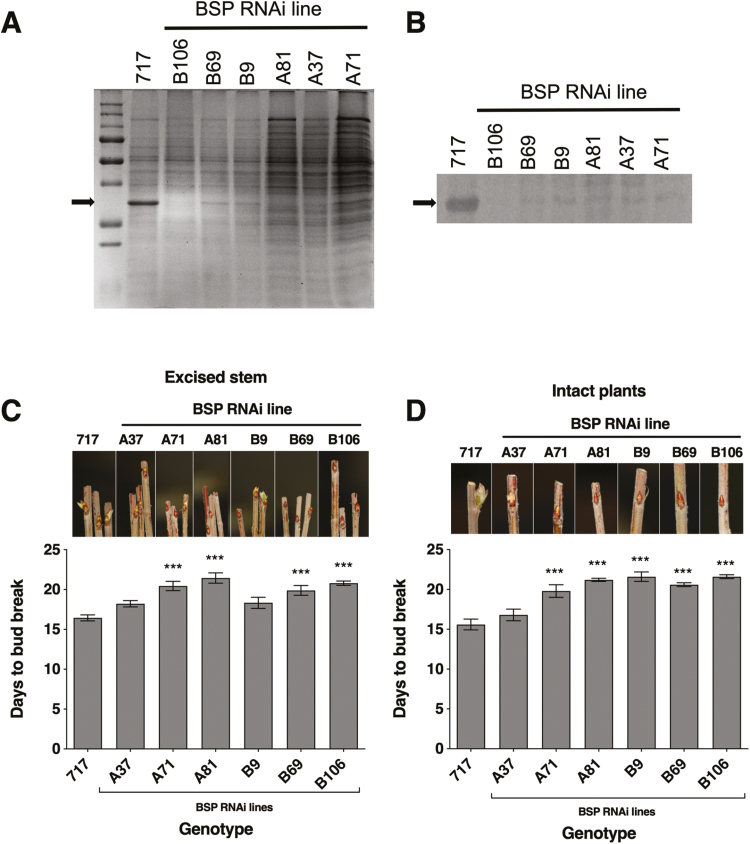
Reduced poplar BSP accumulation delays bud break following dormancy. (A) SDS–PAGE of soluble bark proteins after SD and low temperature treatments and immediately prior to LD treatment at 20 °C; the arrow indicates the location of the 32 kDa BSPs. Each lane represents either control wild-type plants (717) or BSP RNAi lines (A37, A71, A81, B9, B69, and B106) which are independent transformation events where the RNAi construct used for the A and B lines target different regions (see the Materials and methods). (B) Western blot analysis of BSP abundance in the bark of control (717) and BSP RNAi lines after SD and low temperature treatments and immediately prior to LD treatment at 20 °C. Days to visible bud break of control and BSP RNAi lines for (C) excised stems or (D) intact plants after treatment in LDs at 20 °C. Error bars indicate the mean ±SE and *** indicates values significantly different from control at *P*<0.001 based on ANOVA and Dunnett’s multiple comparison tests.

A 103 bp DNA fragment of the *BSPA* gene (*Potri.013G10070*) from *P. trichocarpa* Nisqually was amplified using the forward primer 5'-CCAGAGAATGGAGAGAACTTG-3' and reverse primer 5'-TGGTGATGGGAAGCCAGAAAAC-3', and a 229 bp DNA fragment of the same gene was amplified using the forward primer 5'-TCGCTTAGGGCTTGTTTTTACG-3' and reverse primer 5'-GATGACTCCGTGAATGCTGAATC-3'. The 103 bp fragment starts at nucleotide 507 and ends at nucleotide 610, while the 229 bp fragment starts at nucleotide 132 and extends to nucleotide 360 of the coding sequence. Because of high sequence similarity between *BSPA* and *BSPB*, we predicted that both RNAi constructs would knock down both genes. Purified DNA fragments were cloned into pENTR D-TOPO entry vector using the pENTR™ Directional TOPO Cloning Kit (Invitrogen by Life Technologies). The entry clones were recombined into the binary vector pB7GWIWG2(II) (Karimi *et al.*, 2002) using Gateway LR ClonaseII enzyme mix (Invitrogen by Life Technologies) and verified by DNA sequencing. The destination clone was transformed into *Agrobacterium tumefaciens* strain C58/PMP90 using the heat shock method. Stem sections from *in vitro* plantlets of hybrid poplar (*P. tremula*×*P. alba* clone 717) were transformed by co-cultivation with *A. tumefaciens* strain C58/pMP90 harboring the binary vectors (Leple *et al.*, 1992). Transformed plants were selected on Murashige and Skoog (MS) medium containing 5 mg^–l^ ammonia glufosinate (Basta). Over 40 independent transgenic events from two different RNAi constructs that target either *BSPA* or *BSPB* mRNAs were screened for reduced BSP accumulation after SD-induced dormancy and leaf senescence. This screening identified three events with reduced levels of BSPs for each of the two different *BSP* RNAi constructs (Fig. 1A, B). These six lines were then used in controlled-environment studies to determine the effect of reduced BSP accumulation on growth during LD-mediated N remobilization following dormancy. To reduce confounding effects of shoot competition for stored N, the apical bud and all but a single axillary bud were removed from both excised stems and intact plants prior to growth. In addition, stems were pruned to 30 cm for the excised stem assays, and 50 cm for the intact plants to provide equal amounts of storage tissue between control and BSP RNAi plants.

### Bark microarray analyses

Total RNA from bark samples was purified at the indicated sampling intervals using the Qiagen RNeasy plant mini kit (Qiagen, USA) with modifications and on-column DNA digestion as previously described (Pettengill *et al.*, 2012). RNA quality was assessed using the Experion RNA StdSens Analysis Kit (Bio-Rad Laboratories, CA, USA) and spectrophotometric analysis (Eppendorf BioPhotometer plus; Eppendorf, NY, USA). Biotinylated cRNA was prepared according to the standard Affymetrix protocol (GeneChip 3' IVT expression kit) from 100 ng of total RNA. Following fragmentation, 12.5 μg of cRNA was hybridized for 17 h at 45 °C to GeneChip^®^ Poplar Genome Arrays. GeneChips were washed and stained in the Affymetrix Fluidics Station 450 and scanned using a GeneChip Scanner 3000. Microarray data analysis was completed using R and Bioconductor. Log_2_-transformed expression values were obtained for each probe set. Data filtering involved a similar filtering process based on threshold fractions of ‘present’ calls as described previously (McClintick and Edenberg, 2006), which eliminated probe sets that are unlikely to be reliable while preserving the most significant probe sets. Probe sets that have very low median absolute deviation ≤0.1 were excluded from further analysis. This filtering process resulted in 30 410 probe sets retained for further statistical analysis.

Probe sets that passed the filtering process were statistically analyzed using the limma package (Smyth, 2004) where variances were adjusted by the empirical Bayes (eBayes) method, and adjusted *P*-values for multiple tests were calculated using the Benjamini–Hochberg method (Benjamini and Hochberg, 1995). Probe set annotations were obtained from Affymetrix, and the corresponding *Populus* gene models for each probe set were extracted. For gene models with multiple matching probe sets, the average expression values, average log_2_ ratios for each comparison, and average adjusted *P*-values were calculated based on all matching probe sets. Gene models with an adjusted *P*-value ≤0.01 and a log_2_-fold change ≥1.585 (3-fold change on linear scale) were considered differentially expressed during LD regrowth compared with SD-LT controls.

Pathway analysis was based on the annotated Arabidopsis genome. The corresponding Arabidopsis locus for each *Populus* model was obtained from Phytozome. The mean log_2_ ratios for all the *Populus* models matching single Arabidopsis orthologs were then imported into CIMminer to generate color-coded Clustered Image Maps (CIMs) (‘heat maps’ with the default parameters (https://discover.nci.nih.gov/cimminer/home.do) (Weinstein *et al.*, 1997). Enrichment of Gene Ontology (GO) terms in the up- and down-regulated genes in the differentially expressed genes (DEGs) were investigated through agriGO (Du *et al.*, 2010) where singular enrichment analysis was performed with a hypergeometric test and Yekutieli false discovery rate (FDR; 0.05) under dependency for multiple test adjustment. All microarray data and expression values for all probe sets were submitted to the Gene Expression Omnibus (GEO) at NCBI http://www.ncbi.nlm.nih.gov/geo under the accession number GSE49982.

### Identification of the poplar TAR gene family

Protein sequences were obtained from Phytozome, aligned using ClustalW, and used to construct an unrooted phylogenetic tree with the Neighbor–Joining method (Saitou and Nei, 1987) and a bootstrap value of 10 000 (Felsenstein, 1985) using MEGA7 (Kumar *et al.*, 2016). Evolutionary distances were computed using the Poisson correction method (Zuckerkandl and Pauling, 1965) and are in the units of the number of amino acid substitutions per site. The analysis involved 10 amino acid sequences with all positions containing gaps and missing data eliminated for a total of 114 positions in the final dataset.

### qRT-PCR analysis of gene expression

Total RNA was extracted from a minimum of three biological replicates as previously described (Pettengill *et al.*, 2012). Purified RNA was used for cDNA synthesis according to the manufacturer’s instructions (*iScript*™ cDNA Synthesis Kit, Bio-Rad). The cDNA was used for determination of the relative transcript level of selected target genes using quantitative real-time PCR (qRT-PCR) as previously described (Pettengill *et al.*, 2012). Amplification reactions consisted of 30 s at 95 °C followed by 40 cycles of 5 s at 95 °C and 15 s at 60 °C. Reference gene and relative expression analysis was performed using qbasePLUS as previously described (Pettengill *et al.*, 2012). All qRT-PCR primers for selected target and reference genes are listed in Supplementary Table S1 at *JXB* online and were designed and validated as previously described (Pettengill *et al.*, 2012).

### Protein extraction and analysis

Bark samples were ground using a Freezer/Mill 6970EFM (Spex SamplePrep Metuchen, NJ, USA), and at least three biological replicates were used for protein extraction. Soluble proteins were extracted from bark as previously described (Coleman *et al.*, 1991). Proteins were quantified using the bicinchoninic acid (BCA) assay with minor modifications (Smith *et al.*, 1985; Brown *et al.*, 1989) using a μQuant spectrophotometer (Bio-Tek Instruments, Inc.). Linear regression using Prism software was used to calculate protein concentrations. A 5 μg aliquot of total protein was electrophoresed at 180 V for 1 h by SDS–PAGE in 12% resolving and 4% stacking gels. Gels were stained with Coomassie Blue R-250 and destained with water. Gel images were visualized using a VersaDoc imaging system (Bio-Rad). Protein immunoblots were performed as previously described (Coleman *et al.*, 1991).

### Auxin manipulation experiments

#### Plant material and growth

*Populus trichocarpa* (clone Nisqually) plants were propagated and grown as described in the above ‘Shoot competition experiments during N remobilization’.

#### Bud removal treatments

After completion of the low temperature treatment, plants were prepared by removing all buds except the five uppermost axillary buds and the apical bud, while another set of plants had all axillary and apical buds removed. Both intact plants and plants lacking buds were immediately place in LDs (16 h light/8 h dark) at 20 °C for induction of shoot growth. Bark samples were collected from the same region of the stem at the beginning of the LD treatment (day 0) and after 1, 2, and 3 weeks of LD treatment. At each sampling date, at least three biological replicates were collected. Bark was peeled from stems, immediately frozen in liquid N_2_, and stored in a –80 °C freezer until used for RNA or protein extraction.

#### NPA treatments

1-*N*-naphthylphthalamic acid (NPA) treatments were applied to plants following low temperature treatment to overcome bud dormancy and immediately prior to LD treatment to induce shoot regrowth. The apical bud and the uppermost five axillary buds were left intact while the remaining axillary buds were surgically removed from the stem of plants so that N sink number is consistent between plants. A subset of the plants were treated with either (i) a ring of 50 mM NPA in lanolin paste (Spicer *et al.*, 2013) applied 20 cm below the proximal axillary bud; or (ii) a ring of lanolin paste with 0 mM NPA applied 20 cm below the proximal axillary bud. Plants were randomly assigned to each treatment. NPA (Chem Services, West Chester, PA, USA) was first dissolved in DMSO to a concentration of 0.5 M and then mixed with lanolin paste to give a final concentration of 50 mM. The lanolin paste control (without NPA) contained the same volume of DMSO. The site of application was immediately covered with aluminum foil, plants were transferred to LD greenhouse conditions, and the treatments were reapplied at weekly intervals. Bark tissue was collected from four biological replicates 3 weeks after the start of the LD treatment. For the NPA and lanolin control treatments, bark tissues were subdivided into two sections that included a 20 cm region below the shoot zone but above the site of NPA application, and bark from below the site of treatment. Bark was peeled from stems, immediately frozen in liquid N_2_, and stored in a –80 °C freezer until used for RNA or protein extraction.

#### In vitro auxin treatments

*In vitro* cultured *P. tremula*×*P. alba* hybrid clone INRA 717-1B4 stems were used for auxin treatment as described by Schrader *et al.* (2003). Appropriate 6 cm long and 3 mm thick stems were defoliated and immediately transferred into half-strength liquid Murashige and Skoog (MS) medium lacking auxin. Stem samples were harvested after being incubated at 100 rpm at 20 °C for 0, 2, 4, 8, and 16 h. After 16 h of auxin depletion, 20 μM indole actetic acid (IAA) was added to the medium, and stem samples were harvested after 0.5, 1, 2, and 4 h of IAA treatment. At least three biological replicates were collected at each time point, and all harvested stems were frozen in liquid N_2_ and stored in a –80 °C freezer until used for RNA extraction.

#### Bark auxin measurements

Frozen bark samples were pre-ground and stored at –80 °C prior to analysis. Samples (~20–23 mg per sample) were triple-ground in liquid nitrogen, and 1 ml of 50 mM sodium-phosphate buffer (pH 7.0, containing 1% DETC) was immediately added to each tube. A 1 µg aliquot of [^2^H_3_]tryptophan (d3-Trp, CDN isotopes, Quebec, Canada, #D-7419), 10 ng of [^2^H_5_]indole-3 acetic acid (d5-IAA,#0311532; this and all subsequent compounds were obtained from OlchemIm Ltd, Olomouc, Czech Republic), 2.5 ng of [^2^H_5_]indole-3-[^15^N]acetamide (DN-IAM; #0311541), 5 ng of [^2^H_4_]indole-3-acetonitrile (d4-IAN; #0311851), 2.5 ng of [^2^H_5_]indole-3-acetyl-l-[^15^N]alanine (DN-IAAla; #0311581), 2.5 ng of [^2^H_5_]indole-3-acetyl-l-[^15^N]phenylalanine (DN-IAPhe#0311621), 2.5 ng of [^2^H_5_]indole-3-acetyl-l-[^15^N]valine (DN-IAVal; #0311641), 2.5 ng of [^2^H_5_] indole-3-acetyl-l-[^15^N]leucine (DN-IALeu;t #0311612), 2.5 ng of [^2^H_5_]indole-3-acetyl-l-[^15^N]aspartic acid (DN-IAAsp; #0311592), 2.5 ng of [^2^H_5_]indole-3-acetyl-l-[^15^N]glutamic acid (DN-IAGlu; #0316132), and 2.5 ng of [^2^H_5_]indole-3-acetyl-l-[^15^N]tryptophan (DN-IATrp; #0311632) were added into each tube as internal standards (ISTD). Samples were vortexed, extracted for 20 min at 4 °C on a lab nutator, and centrifuged at 12 000 *g* for 15 min at 4 °C. Supernatants were collected, and the pH was adjusted to 3 using 1 N HCl. Auxinic compounds were further purified and concentrated by passing each supernatant over an HLB column [conditioned using 1 ml of methanol (LC-MS/MS grade, Fisher Scientific, Pittsburgh, PA, USA #A456-1) followed by 1 ml of water and 0.5 ml of 50 mM Na-phosphate buffer, pH 2.7]. After sample loading, HLB columns were washed with 2 ml of 5% methanol and finally eluted with 2 ml of 80% methanol. Eluates (containing auxins and auxin metabolites) were dried under N gas, re-dissolved with 1.00 ml of methanol, and filtered through 4 mm 0.2 µm PTFE filters (Phenomenex, Inc., Torrance, CA, USA, #AF0-3202–52). A 0.2–0.5 µl volume of each sample was injected for LC-MS/MS analyses conducted using an Agilent 6460 triple quadrupole (QQQ) LC-MS/MS system (Agilent Technologies, Santa Clara, CA, USA), as described previously (Blakeslee and Murphy, 2016; Zhang *et al.*, 2016). Compound identities were confirmed by comparing mass transitions and retention times with those of authentic standards, as in Zhang *et al.* (2016). Compounds were quantified using linear concentration curves generated with authentic standards and the Agilent MassHunter Quantitative Analysis software package (Agilent Technologies), as described in Blakeslee and Murphy (2016).

### ^13^N transport study

#### Plant materials and growth conditions

Wild-type (WT) and BSP RNAi poplars of *P. tremula*×*P. alba* clone 717 were used in the experiments. *In vitro* propagated 7- to 8-week-old plantlets were transplanted into potting soil (Professional growing mix, Sungro^®^) in plastic pots (8 cm×8 cm×7 cm). After transplanting, plants were grown in a plant growth chamber (A1000, Conviron) under an LD photoperiod (16 h light, 24 °C daytime, 22 °C dark) for 5 weeks and then a subset of the plants were used for ^13^N experiments. After the LD plants were removed, the remaining plants were exposed to an SD photoperiod (10 h light, 24 °C daytime, 22 °C dark) for 6 weeks, and then the SD treatment was supplemented with cold treatment (SD-LT) (10 h photoperiod, 12 °C daytime, 7 °C dark) for 3 weeks before SD-LT ^13^N experiments. At each time point, 10 poplar plants of each genotype that were similar in size were chosen for experiments.

#### ^13^N labeling

Each plant was transferred to a growth chamber containing the positron emission tomography (PET) scanner and positioned at the center of the PET field of view (Wang *et al.*, 2014). A single load leaf at a leaf plastochron index (LPI) of 10–12 was clamped inside a transparent cuvette, lit by growth chamber lamps. ^13^N was produced by bombarding a water target in a cyclotron to produce [^13^N]NH_3_. The ^13^NH_3_ solution was basified with KOH and injected into the cuvette onto an absorbent pad at the bottom of the cuvette. The delivered activity was measured in a dose calibrator/gamma radiation well counter (CRC-258, Capintec, Florham Park, NJ, USA) (~0.16–0.3 MBq per dose), and the time was recorded for decay correction (half-life of ^13^N=9.965 min). A pump was attached to the cuvette outlet to provide airflow through the cuvette. The leaf was treated for 15 min before removing the cuvette, and then the PET scanned continuously for 1 h. Plants were taken out from the scanner, dissected into four parts: upper stem including the apex; lower stem; petiole and the half of the load leaf not in the cuvette; and the load zone, which is the part of the leaf in the cuvette. The radioactivity of each plant part was measured in a calibrated gamma radiation well counter (Shielded Well Gamma Sample Counter Head: Model 203, NATS Inc., Middletown, CT, USA; Scaler/Ratemeter Model 2200, Ludlum Measurements, Inc., Sweetwater, TX, USA) and the time was recorded for decay correction.

To test how much of the ^13^N in the leaf had been assimilated, a piece of the load zone was excised and the radioactivity was measured before and after volatilization of ^13^NH_3_, as described by Hanik *et al.* (2010). Briefly, the piece of leaf was ground in 1 M KOH solution to raise the pH and convert any ^13^NH_4_^+^ to the volatile form ^13^NH_3_, and the solution was sparged with compressed air for 20 min to remove any ^13^NH_3_. After 5, 10, and 20 min of sparging, we found no decrease in the decay-corrected radioactivity, indicating that there was no free ^13^NH_3_/^13^NH_4_^+^ in the leaf by the end of the 1 h plant incubation.

^13^N export was calculated as a percentage of total ^13^N in the plant, after decay correction. Partitioning to the lower stem was calculated using the activity measured in the lower stem compared with the total activity detected in plants. Total export was calculated using the combined activity of apex/upper stem and lower stem as a percentage of the total activity in the plant.

#### Growth measurements

Growth measurements were taken 3 d before the first day of each set of ^13^N experiments, including stem height, and total number of leaves (upper leaves with length >2 cm were counted; lower leaves with length >7 cm were counted). Load leaf length (cm) from tip to petiole was measured, as well as width (cm) at the widest part of the leaf. Load leaf average chlorophyll content (mg m^–2^) was measured *in vivo* using a chlorophyll fluorescence meter (CCM-300, OPTI-SCIENCES, Hudson, NH, USA), based on the method of Gitelson *et al.* (1999). Chlorophyll measurements were taken prior to ^13^N experiments at three different positions on each leaf and averaged for the leaf ^13^N labeling.

### Statistical analysis

At least three biological replicates were used in each experiment. ANOVA and mean separation tests were performed using Prism 8 software (GraphPad Software LLC).

## Results

### Nitrogen availability impacts leaf area, sylleptic shoot production, and stem growth

Prior to examining how levels of BSP accumulation or shoot N sink competition influence growth, we first examined the relationship between N availability and growth of *P. tremula*×*P. alba* clone 717-1B4 (WT-717) since this clone was used in the transgenic studies. Similar to previous studies using a different poplar genotype (Cooke *et al.*, 2005), increased N availability results in increased leaf area, greater sylleptic shoot production, and increased stem diameter (Supplementary Fig. S1A–F). Sylleptic shoot production appeared to have a threshold N level above which shoot production was significantly enhanced (Supplementary Fig. S1C, E).

### Reduced BSP accumulation impacts shoot growth following dormancy

**Fig. 2.**
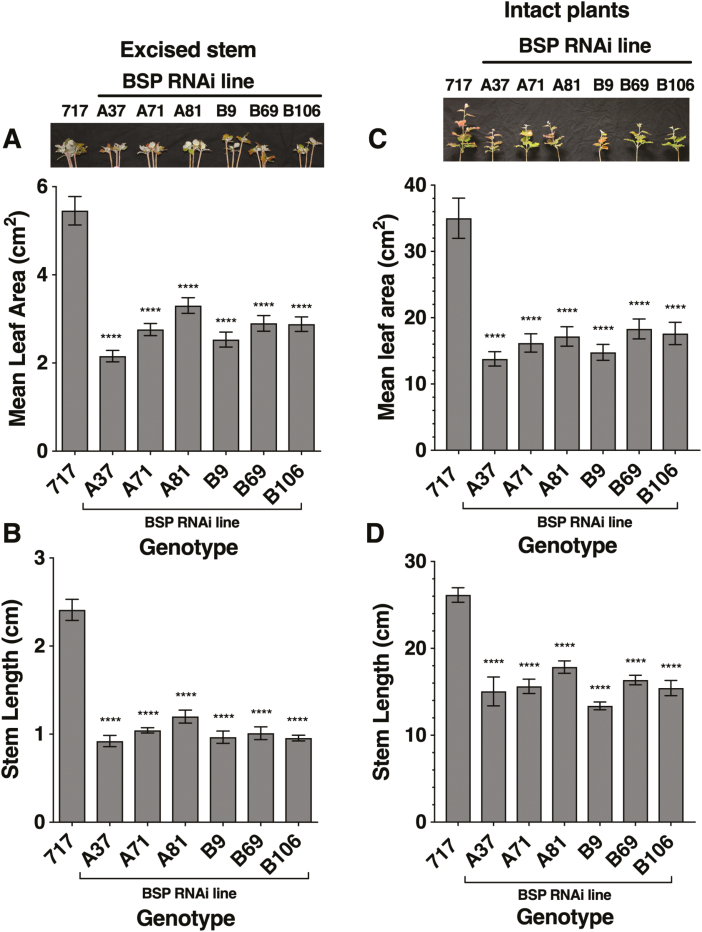
Reduced BSP accumulation via RNAi knockdown reduces growth following bud break. (A) Mean total leaf area per stem and (B) length of new stem growth 6 weeks after bud break of excised stems for control (717) and BSP RNAi lines (A37, A71, A81, B9, B69, and B106) and (C) mean total leaf area per stem and (D) length of new stem growth 6 weeks after bud break for intact plants. Each BSP RNAi line is an independent transformation event where the RNAi constructs for the A and B lines target different regions. Error bars indicate the mean ±SE and **** indicates values significantly different from control at *P*<0.0001 based on ANOVA and Dunnett’s multiple comparison tests.

Unexpectedly, bud break was delayed in four of the six RNAi events with excised stems (Fig. 1C) and in five of the six RNAi events with intact plants (Fig. 1D), suggesting that N storage levels influence bud expansion and bud break. Leaf area and stem growth were measured as outputs of N status after 6 weeks of growth following bud break (adjusted for differences in timing of bud break among BSP RNAi lines) using both excised stems and intact plants. After 6 weeks of growth, a 40–60% reduction in leaf area for excised stems (Fig. 2A) and a 50–60% reduction for intact plants (Fig. 2C) occurred in the BSP RNAi plants compared with controls. Similar to leaf area, stem growth was also reduced in BSP RNAi plants by ≥50% for both excised stems and intact plants (Fig. 2B, D). Similar reductions in leaf dry weight (Supplementary Fig. S2B, F), stem dry weight (Supplementary Fig. S2C, G), and shoot biomass (Supplementary Fig. S2D, H) were also observed for all of the BSP RNAi lines.

A second approach manipulated shoot sink demand for N, while leaving N storage levels unchanged (see the Materials and methods for details). Increasing sink demand by increasing competition from one to three shoots reduced leaf area by 43% (107.2 cm^2^ versus 61.41 cm^2^) (Supplementary Fig. 3SA) and continued increases in sink number (6, 9, or 12 shoots) further decreased leaf area, with the greatest reduction (70%) occurring in plants with 12 axillary shoots. A similar trend was observed for stem length (Supplementary Fig. S3B), although differences were only significant for plants with one, three, or six shoots compared with plants with nine or 12 shoots. The number of sylleptic shoots developing from regrowing shoots was also related to sink competition (Supplementary Fig S3C) since the greatest number of sylleptic shoots (9.8) were produced from plants with a single regrowing shoot, while significantly fewer sylleptic shoots were produced from plants with six, nine, or 12 growing shoots (Supplementary Fig. S3C). Similar trends of reduced leaf area, stem length, and sylleptic shoot number also occurred when sink competition between sylleptic shoots formed during the previous growth cycle was increased (Supplementary Fig. S3D–F).

Competition between sylleptic and proleptic shoots was examined by varying the ratios of proleptic and previously formed sylleptic shoots and then measuring growth following dormancy (Supplementary Fig. S4A–E). No differences in leaf area or stem growth for shoots arising from axillary buds occurred when sylleptic shoot competition increased from one to 10 shoots; however, growth was significantly reduced by competition from increased axillary shoot numbers (1 versus 10) (Supplementary Fig. S4A, B). Increased competition from axillary shoots also reduced the production of new sylleptic shoots from shoots growing from axillary buds (Supplementary Fig. S4C). Both leaf area and stem length were significantly reduced for shoots arising from sylleptic shoots when the number of shoots from axillary buds was increased from one to 10 shoots or when the sylleptic shoot number was increased (Supplementary Fig. S4D, E). Therefore, it appears that competition for stored reserves depends upon the shoot type.

### BSP accumulation contributes to sink demand and N export from leaves during autumn

No significant differences in plant height, number of leaves, leaf area, and chlorophyll content were observed between WT and BSP RNAi plants used for ^13^N transport studies (Supplementary Fig. S5). The plants continued to grow substantially in height after switching from LDs to SDs (54% increase for WT, and 67% for BSP RNAi) and produced 3–4 additional leaves. By the sixth week of SD treatment, plants stopped growing in height, stopped producing new leaves, and formed a terminal bud. Leaf senescence occurred upon exposure to low temperature, resulting in similar reductions in chlorophyll content of both WT and BSP RNAi plants (Supplementary Fig. S5D).

**Fig. 3.**
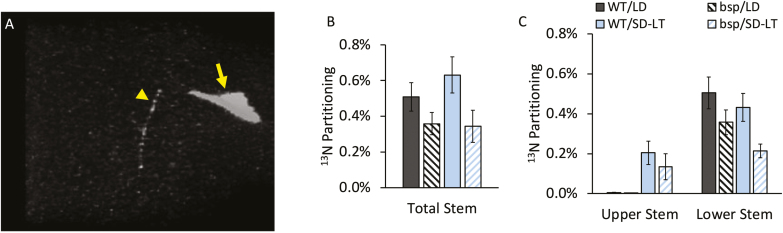
^13^N partitioning in poplars during SD-induced BSP accumulation. (A) PET image of ^13^N distribution in a representative poplar plant. ^13^NH_3_ was fed to the load zone (yellow arrow), the portion of the load leaf clamped inside a leaf cuvette, and the plant was imaged 1 h later. The arrowhead points to ^13^N labeling in the stem. (B) ^13^N partitioning in wild-type (WT) and BSP RNAi (*bsp*) poplar stems, and (C) ^13^N partitioning separated into the regions of the stem below the load leaf (lower stem) and above the load leaf (upper stem). Plants were grown in a long-day photoperiod (LD), or grown first in LDs followed by 6 weeks in a short-day photoperiod plus another 3–5 weeks in SDs and low temperature (SD-LT). Bars indicate the mean ±SE ^13^N partitioning to the stem as a percentage of total ^13^N in the plant.

Comparison of N partitioning between the WT and a BSP RNAi line during SD-induced BSP accumulation was examined using ^13^N labeling and PET. When ^13^NH_3_ is fed to leaves, it is quickly assimilated into amino acids, presumably through the GS/GOGAT cycle (Hanik *et al.*, 2010) and all of the ^13^NH_3_ in the poplar leaves had been assimilated by 1 h of incubation (see the Materials and methods). A relatively small portion of ^13^N was exported from the leaf during the 1 h incubation period of the experiments. Most of the exported ^13^N was detected in the upper part of the lower stem (Fig. 3A). Based on previous experiments with carbon-11 (^11^C), 1 h is sufficient time for the phloem transport stream to reach the roots of small poplar plants like these (Babst *et al.*, 2005), suggesting that N is selectively removed from the phloem for use or storage in the stems of poplars.

Total partitioning of ^13^N to the stem was lower in BSP RNAi than in WT poplars (Fig. 3B). Partitioning of ^13^N to the upper stem increased substantially after SD and low-temperature treatment (SD-LT) (Fig. 3C), but the total export did not change significantly. In the BSP RNAi line, partitioning of ^13^N to the lower stem decreased after SD-LT treatment. Also, the ^13^N partitioning to the lower stem became significantly less in BSP RNAi plants compared with the WT after SD-LT treatment.

### BSP catabolism and N remobilization involve extensive changes in bark gene expression

DNA microarrays were used to identify DEGs during N remobilization (log_2_-fold >1.5 and adjusted *P*-value ≤0.01) by comparing gene expression levels in the bark of plants after 1, 2, or 3 weeks of LD growth with those of the bark of plants that had received sufficient chilling to overcome dormancy but just prior to transfer to LD conditions. After 1 week of LD growth, a total of 2227 genes were differentially expressed, with three times as many genes exhibiting increased expression compared with reduced expression (Supplementary Fig. S6). After 3 weeks of LD growth, the number of DEGs increased to 5643, with twice as many genes showing increased expression compared with reduced expression (Supplementary Fig. S6). GO analysis of DEGs (Supplementary Table S1) revealed enriched biological process ontologies related to receptor-linked protein signaling, transport, cellular response to hormone stimulus, and hormone-mediated signaling pathways, while enriched functional processes included catalytic, transporter, and peptidase activity. MapMan analysis mapped DEGs to various processes, and 25% of genes that mapped to hormone BINS (Bin 17; Supplementary Table S2) included auxin-related process (42 of 168 genes).

### Auxin-related gene expression is up-regulated during N remobilization

Because of the enrichment for auxin-related genes, the microarray data were queried for expression of genes encoding auxin transporters (*AUX/LAX*, *PIN*, and *ABCB*), and auxin signaling and metabolism (*Aux-IAA*, *ARF*, and *GH3*) during LD-associated BSP catabolism and N remobilization (Supplementary Fig. S7). Except for one auxin transporter (*Potri.008G050400*; *ABCB18*), all differentially expressed auxin transporter genes showed increased expression, at least transiently, during LD-induced BSP catabolism and N remobilization (Supplementary Fig. S7). Two putative auxin transporter genes, *Potri.005G187500* (*Aux7/Lax8*) and *Potri.009G132100* (*Aux6/Lax3*), clustered together and had the greatest induction during LD-mediated N remobilization (Supplementary Fig. S7A). The majority of differentially expressed Aux/IAA repressor genes were also induced during LD treatment (Supplementary Fig. S7B), and one cluster of eight Aux/IAA genes (*IAA3.2*, *IAA12.1*, *IAA12.2*, *IAA19.3*, *IAA20.1*, *IAA26.2*, *IAA27.2*, and *IAA28.2*) was highly induced during renewed shoot growth (Supplementary Fig. S7B) while only two genes (*Potri.008G161100*; *IAA3.5* and *Potri.018G127800*; *IAA29.2*) showed reduced expression. Auxin response factors (ARFs) clustered into three major groups (Supplementary Fig. S7C): nine genes were up-regulated; six genes were down-regulated; and six genes showed little change in expression during LD treatment. Although the *GRETCGEN HAGEN 3* (*GH3*) auxin aminotransferase gene expression patterns were clustered into two major groups, all of the genes showed a generalized pattern where expression increased after 2 weeks of LD treatment and then declined in week 3. *GH3-9* (*Potri.002G206400*) and *GH3-5* (*Potri.011G129700*) clustered together and showed the greatest increase in expression after 2 weeks of LD treatment.

### Aspartic and serine protease gene expression is up-regulated during N remobilization

The poplar genome is estimated to contain 955 genes encoding proteases (García-Lorenzo *et al.*, 2006), and DNA microarray analysis of gene expression during LD-induced N remobilization showed that a large number of protease genes are expressed in bark but the majority showed either little change or a decline in transcript abundance during LD-induced N remobilization (Supplementary Fig. S8). A cluster of 37 putative protease genes showed increased transcript abundance during N remobilization (Supplementary Fig. S8), and within this larger cluster was a six gene cluster that included four putative aspartic proteases (*Potri.001G028200*, *Potri.002G104600*, *Potri.015G051800*, and *Potri.018G014600*) and two putative serine proteases (*Potri.002G120400* and *Potri.009G133400*) that were highly induced (log_2_-fold increase ranged from 2.1 to 7.5) and may represent proteases that play possible roles in BSP catabolism.

### Amino acid transporter gene expression is up-regulated during N remobilization

Changes in amino acid transporter expression (Supplementary Fig. S9), including the *ATF-AAP* superfamily (Supplementary Fig. S9A), *PTR-NRT* family (Supplementary Fig. S9B), *APC* family (Supplementary Fig. S9C), *UmaniT* family (Supplementary Fig. S9D), and the *OPT* family (Supplementary Fig. S9E), during N remobilization were also examined using DNA microarrays. In general, a greater proportion of genes belonging to the *ATF-AAP* and *APC* families showed increased expression levels during N remobilization, while a greater proportion of members of the *PTR-NRT* and *OPT* families appeared to be repressed during N remobilization (Supplementary Fig. S9). In the *ATF-AAP* family, a cluster of four genes (*Potri.010.G128300*, *Potri.005G174000*, *Potri.005G181600*, and *Potri.009G132100*) were highly induced and showed a steady increase in expression during the 3 weeks of LD treatment that ranged between a log_2_-fold increase of 4.8 to 7.6 (Supplementary Fig. 9SA). The *APC* family also included a cluster of eight genes whose expression increased during LD treatment (Supplementary Fig. S9C), with increases ranging from a log_2_-fold increase of 0.68 for *Potri.001G378500* to 2.4 for *Potri.01G24100* (*CAT10*).

### Auxin biosynthesis and transport are associated with BSP catabolism

**Fig. 4.**
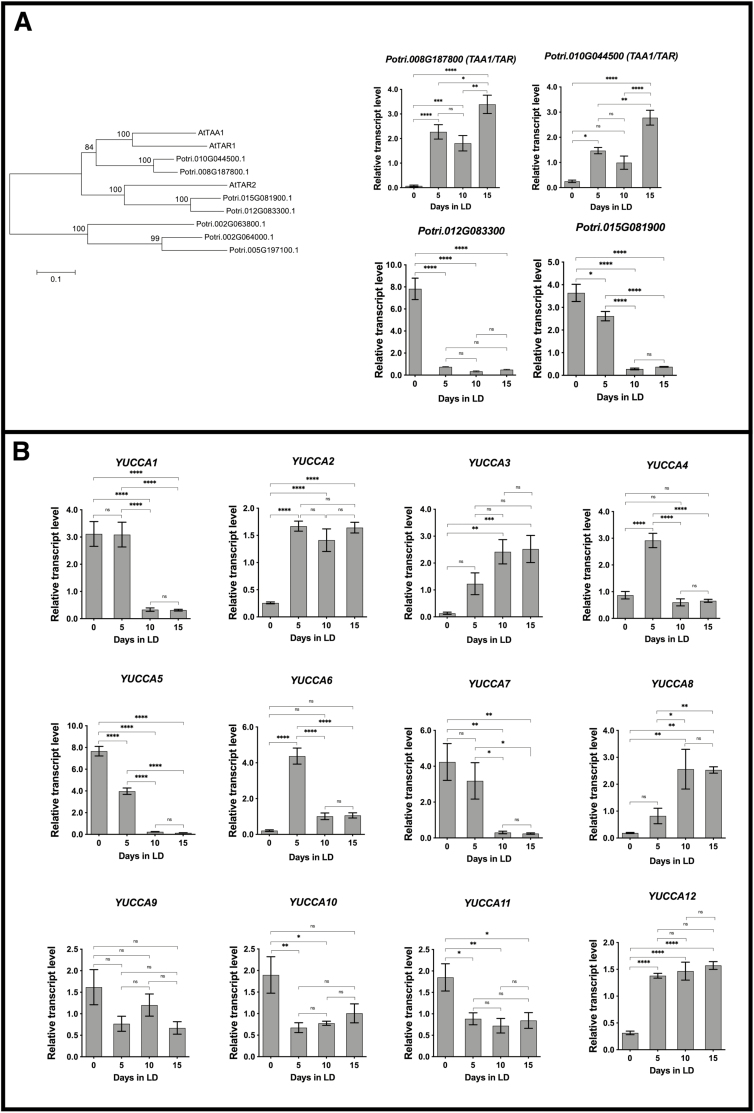
Expression of auxin biosynthesis genes in expanding buds and shoots of poplar. Relative expression of (A) TAA1/TAR and (B) YUCCA genes was determined by qRT-PCR. Plants (*Populus trichocarpa*, Nisqually) were first grown in LDs for ~6 weeks followed by SD treatment for 8 weeks at 20 °C to induce dormancy. Leaf senescence and abscission were then induced with 6 weeks of further SDs at low temperature (LT) (10 °C, light; 4 °C dark). After leaf senescence and abscission, the plants were placed in the dark at 4 °C for 7 weeks to release buds from dormancy. Renewed shoot growth was then initiated by placing plants in LDs at 20 °C. Three independent replicates were collected for RNA purification at the start of the LD treatment and at 5 d intervals for 15 d. Error bars indicate the mean ±SE, and values significantly different from day 0 are indicated by *P<0.05, **P<0.01, ***P<0.001, and ****P<0.0001 based on ANOVA and Tukey’s multiple comparison tests.

Because microarray results suggested that auxin transport and signaling could have a role in N remobilization, the expression of genes involved in auxin biosynthesis in expanding buds and shoots (Fig. 4) was examined. Four poplar homologs to *TAA1/TAR* genes (Fig. 4A) were identified, and expression of two of these genes (*Potri.008G187800* and *Potri.010G044500*) increased in expanding buds and shoots during LD N remobilization, while expression of the other two genes declined. Expression of six (*YUCCA2*, *YUCCA3*, *YUCCA4*, *YUCCA6*, *YUCCA8*, and *YUCCA12*) of the 12 poplar *YUCCA* genes (Ye *et al.*, 2009) increased during N remobilization, while expression of the remaining six genes declined (Fig. 4B).

**Fig. 5.**
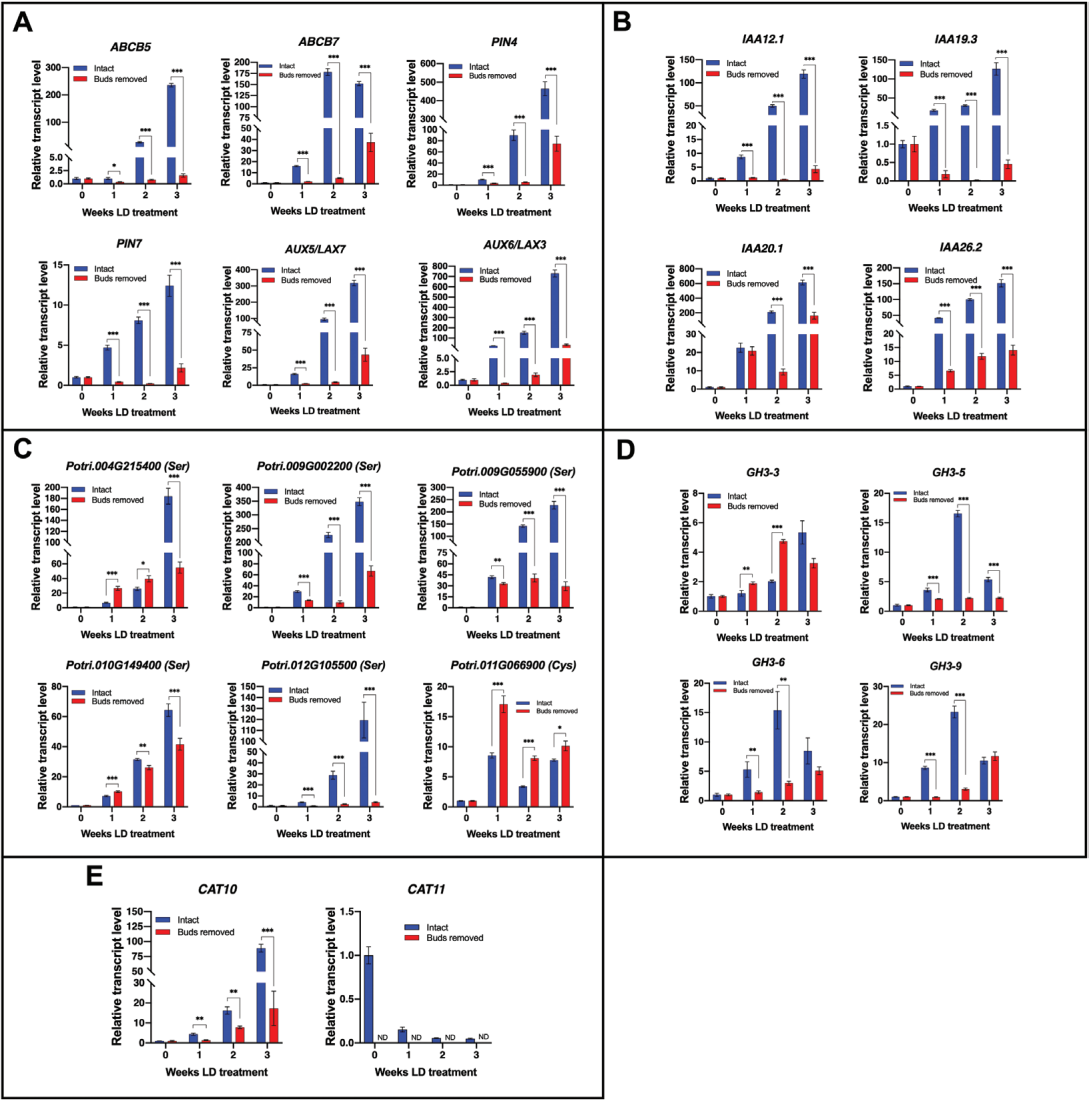
Effect of bud removal on bark gene expression during growth following dormancy. The relative expression determined by qRT-PCR of selected (A) auxin transporter genes, (B) AUX/IAA signaling genes, (C) protease genes, (D) auxin-responsive GH3 genes, and (E) the amino acid transporter genes *CAT10* and *CAT11* for intact plants (blue bars) and plants with buds removed (red bars). Plants (*Populus trichocarpa*, Nisqually) were first grown in LDs for ~6 weeks followed by SD treatment for 8 weeks at 20 °C to induce dormancy. Leaf senescence and abscission were then induced with 6 weeks of further SDs at low temperature (LT) (10 °C, light; 4 °C dark). After leaf senescence and abscission, the plants were placed in the dark at 4 °C for 7 weeks to release buds from dormancy. Prior to placing plants in LDs at 20 °C to initiate bud break and shoot growth, buds were removed from a set of plants and another set was left intact. At least three independent bark replicates were collected for RNA purification at the start of the LD treatment and at weekly intervals for 3 weeks. Error bars indicate the mean ±SE, and ND indicates not detected; values significantly different between intact and plants lacking buds are indicated by *P<0.05; **P<0.01, and ***P<0.001 based on multiple *t*-tests and Holm–Sidak multiple comparison test.

Since shoot growth is required for BSP degradation and genes involved in auxin production are expressed in expanding buds and shoots following dormancy, it was determined whether removal of the source of auxin (i.e. buds) would affect bark expression of genes during BSP catabolism and N remobilization. Based on microarray expression patterns, a set of auxin transporter, *Aux/IAA*, *GH-3*, protease, and amino acid transporter genes were selected to determine if their expression differed between intact plants and those whose buds were removed after treatment with low temperature to overcome dormancy prior to LD treatment. Expression of auxin transporter genes increased after 1 week of LD treatment and continued to increase during the 3 week treatment interval in intact plants (Fig. 5A). In plants lacking buds, auxin transporter gene expression also increased, but to a much lesser extent, and the magnitude of the increase was significantly reduced at each weekly time point compared with intact plants (Fig. 5A). Similar to auxin transporter gene expression, *AUX/IAA* gene expression also increased steadily during LD treatment in intact plants, but was significantly reduced in plants lacking buds compared with intact plants (Fig. 5B).

Selected protease genes showed a similar expression pattern to that of auxin transporter and *AUX/IAA* genes during the 3 week LD treatment, but the differences between intact plants and those lacking buds were more variable (Fig. 5C). Three of the selected protease genes (*Potri.009G002200*, *Potri.009G055900*, and *Potri.012G105500*) were expressed at much greater levels throughout the 3 week regrowth period in intact plants compared with plants lacking buds. Expression of *Potri.004G215400* and *Potri.010G149400* was initially greater in plants lacking buds after 1 and 2 weeks, but after 3 weeks of growth bark expression in intact plants was significantly greater than in plants with buds removed. A putative cysteine protease (*Potri.011G066900*) showed a different pattern, with greater expression in plants lacking buds compared with intact plants during the LD treatment.

Expression of auxin-induced *GH3* genes also differed between intact plants and plants lacking buds (Fig. 5D). In intact plants, expression of *GH3-5*, *GH3-6*, and *GH3-9* peaked after 2 weeks of LD treatment and declined by the third week, while in plants lacking buds the expression of these three genes showed a gradual increase in LDs but the levels were reduced compared with intact plants. For *GH3-3*, expression tended to be greater in the bark of plants lacking buds compared with intact plants (Fig. 5D).

The amino acid transporter genes *CAT10* and *CAT11* showed contrasting patterns of gene expression, where *CAT10* expression increased during the 3 week LD treatment in both intact plants and plants lacking buds but the transcript abundance was significantly greater in intact plants (Fig. 5E), whereas *CAT11* transcript abundance declined during LD treatment in intact plants and was not detected in plants lacking buds.

### Blocking auxin transport inhibits BSP catabolism and N remobilization

The possible role of auxin in regulating BSP catabolism and N remobilization was further investigated using a physiological approach by manipulating auxin transport using the auxin efflux inhibitor NPA to inhibit or block polar auxin transport and then assessing BSP catabolism and expression of genes associated with N remobilization. NPA applied to the stem in a ring of lanolin at the concentration (50 mM) used by Spicer *et al.* (2013) to inhibit poplar auxin transport in poplar consistently inhibited BSP catabolism below the site of NPA application after 21 d of growth following dormancy (Supplementary Fig. S10). LC-MS/MS was used to quantify bark IAA, IAA-Ala, and NPA concentrations after 21 d of treatment at the various NPA concentrations (Supplementary Fig. S11A–C). These measurements revealed a consistent pattern of reduced bark IAA levels below the site of treatment compared with bark above the treatment site, irrespective of NPA concentration. However, 50 mM NPA was required to achieve a significant reduction in bark IAA content below the site of treatment (Supplementary Fig. S11A). No significant differences were detected between bark tissue above and below the site of treatment across the various NPA concentrations for IAA-Ala levels (Supplementary Fig. S11B), and this general pattern was also observed for NPA levels (Supplementary Fig. S11C). We did find that, after 21 d of 25 mM and 50 mM NPA treatment, Trp levels in bark were significantly greater in bark below the site of treatment (Supplementary Fig. S11D).

**Fig. 6.**
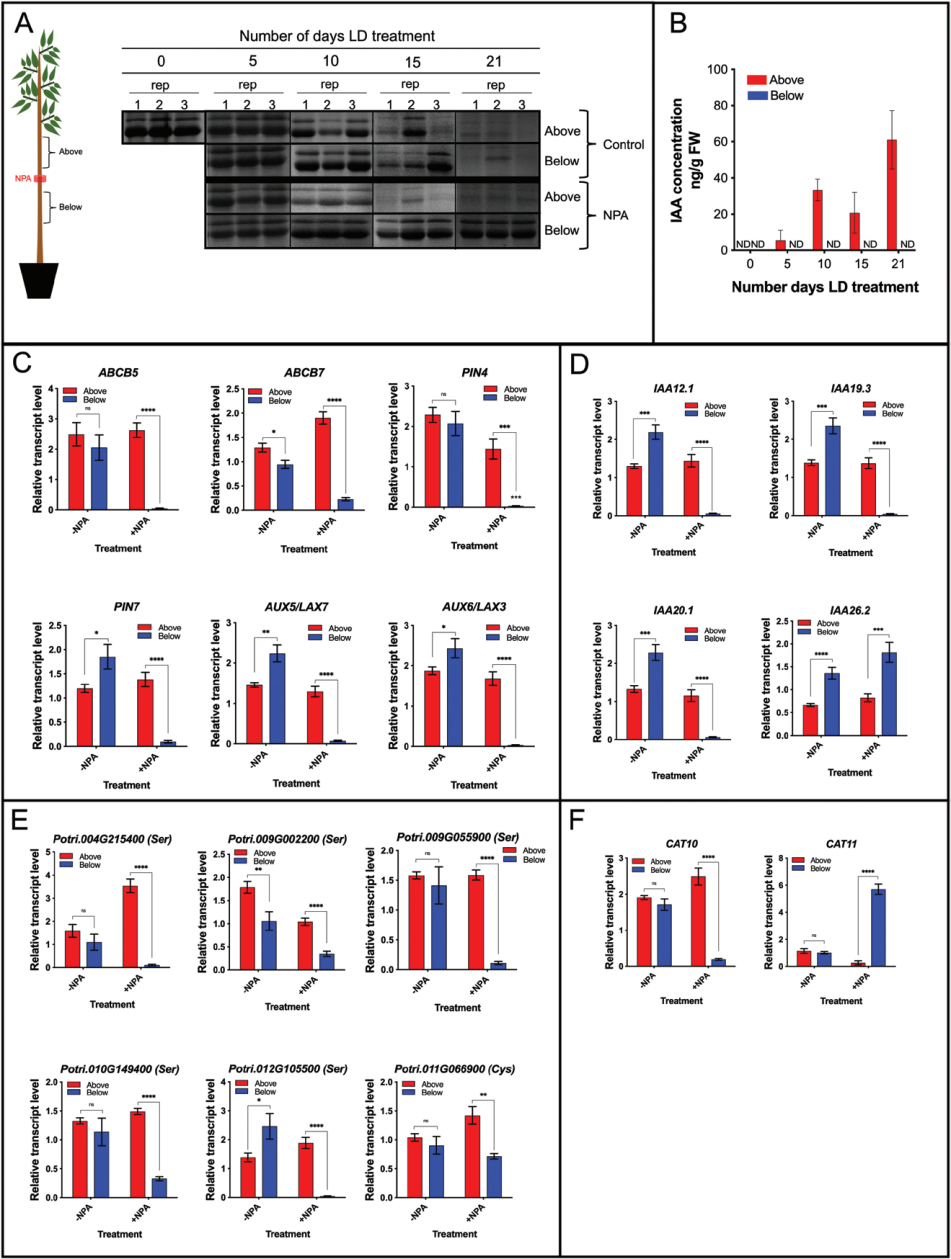
Inhibiting auxin transport impacts bark gene expression during growth following dormancy. (A) Diagram of NPA treatments and SDS–PAGE analysis of BSP abundance above and below the site of stem ringing with lanolin paste containing either 0 or 50 mM NPA during LD-mediated BSP catabolism and N remobilization. (B) IAA levels measured by LC-MS/MS in bark above and below the site of NPA treatment during the LD treatment. Relative gene expression determined by qRT-PCR in bark of plants (*Populus trichocarpa*, Nisqually) above and below the site of either 0 mM (–NPA) or 50 mM (+NPA) NPA treatments of (C) auxin transporter genes, (D) AUX/IAA signaling genes, (E) protease genes, and the amino acid transporter genes *CAT10* and *CAT1,* after 3 weeks of LD treatment. Plants were first grown in LDs for ~6 weeks followed by SD treatment for 8 weeks at 20 °C to induce dormancy. Leaf senescence and abscission were then induced with 6 weeks of further SDs at low temperature (LT) (10 °C, light; 4 °C dark). After leaf senescence and abscission, the plants were placed in the dark at 4 °C for 7 weeks to release buds from dormancy. Prior to placing plants in LDs at 20 °C to initiate bud break and shoot growth, all buds except the apical and uppermost five axillary buds were removed and the stem was ringed at its midpoint with lanolin paste containing 50 mM NPA or lanolin lacking NPA. At least three independent bark replicates were collected for RNA purification after 3 weeks of LD treatment. Error bars indicate the mean ±SE; ND indicates below the detection limits; values significantly different between bark above or below the site of NPA treatment are indicated by *P<0.05; **P<0.01, ***P<0.001, and ****P<0.0001 based on multiple *t*-tests and Holm–Sidak multiple comparison test.

Based on these initial studies, poplar stems were treated with 50 mM NPA prior to regrowth following dormancy, and differences in BSP abundance were observed within 15 d of LD treatment between bark located above and below the site of NPA treatment, while control treatments showed no difference (Fig. 6A). After 21 d of regrowth, little if any BSP was detected in bark above the site of NPA treatment while BSP levels below the site of treatment were similar to those at the beginning of the LD treatment (Fig. 6A). By 5 d of LD treatment, IAA levels increased in bark above the site of NPA treatment and after 21 d bark IAA levels above the site of treatment averaged 61 ng g^–1^ FW while IAA was below the detection limit for our LC-MS/MS analyses in bark below the site of NPA treatment at all sample times (Fig. 6B).

Expression of selected genes (based on microarray analysis) involved in auxin transport and signaling, genes encoding proteases, as well as the amino acid transporter-encoding genes *CAT10* and *CAT11* were used to determine if they were differentially expressed in response to NPA treatment. As shown in Fig. 6C, transcript levels for the selected auxin transporters were significantly reduced in bark below the site of NPA treatment compared with bark above the treatment site. Similar to auxin transporters, differential expression was also observed between bark locations for transcripts of three *AUX/IAA* (*IAA12.1*, *IAA19.3*, and *IAA201*) genes while one gene, *IAA26.2*, showed increased expression in bark below the site of NPA treatment (Fig. 6D). Interestingly, in control/mock-treated plants (ringed with lanolin paste lacking NPA), expression of the selected *AUX/IAA* genes was greater in bark below the treatment site (Fig. 6D). Protease gene expression also showed a differential pattern of expression between bark above and below the location of NPA application (Fig. 6E). Expression of the amino acid transporter genes *CAT10* and *CAT11* differed when polar auxin transport was blocked with NPA. *CAT10* expression was significantly reduced in bark below the site of NPA treatment, while *CAT11* was induced (Fig. 6F).

### Protease genes associated with N remobilization are auxin inducible

**Fig. 7.**
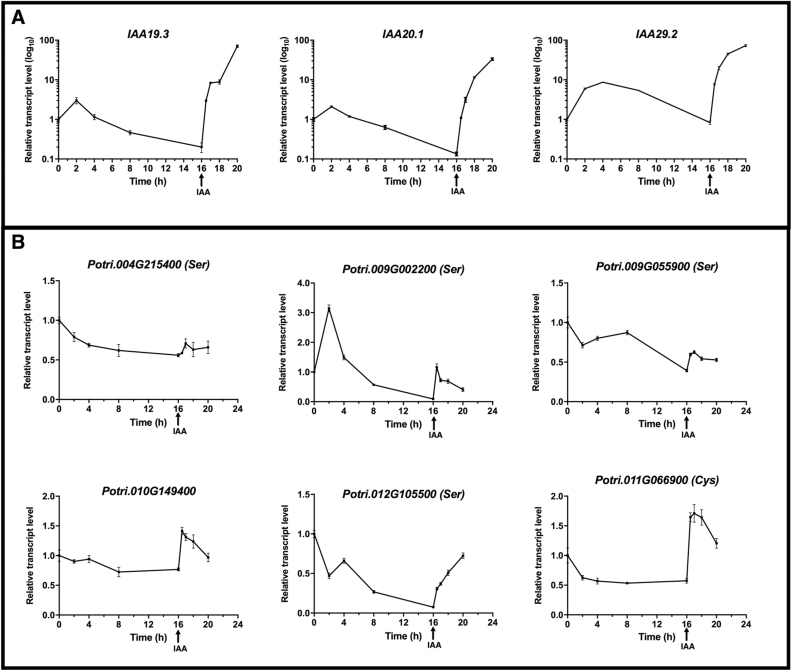
Auxin responsiveness of poplar protease genes. *In vitro* grown defoliated stems of poplar (*P. tremula*×*P. alba* hybrid clone INRA 717-1B4) were incubated in LS growth medium lacking hormones for 16 h to deplete endogenous auxin. After 16 h of depletion, 20 μM IAA was added and incubation was continued for 4 h. Stems were collected at the indicated time points and the relative expression of (A) auxin-responsive AUX/IAA genes and (B) protease genes was determined by qRT-PCR. At least three biological replicates were collected at each time point, and error bars indicate the mean ±SE.

To determine if selected protease genes were auxin inducible, stems of *in vitro* cultured poplars were first incubated in growth medium lacking auxin to deplete endogenous IAA for 16 h followed by treatment with IAA as previously described (Schrader *et al.*, 2003). Throughout the depletion and IAA treatment periods, transcript levels of the same set of protease genes that were used in the NPA and bud removal experiments were assayed. Concurrently, the expression of auxin-inducible *AUX/IAA* genes was determined and showed that transcript levels steadily declined over the 16 h depletion period (Fig. 7A). With the addition of 20 μM IAA to the growth medium, transcript levels of the *AUX/IAA* genes rapidly increased several-fold (Fig. 7A), demonstrating auxin-inducible expression in this assay system.

Protease gene expression showed a general decline during auxin depletion; however, the extent of the decline varied between genes (Fig. 7B). For half of the genes (*Potri.004G215400*, *Potri.010G149400*, and *Potri.011G066900*) the decline was gradual and stabilized after ~4 h of auxin depletion. The remaining three genes (*Potri.009G002200*, *Potri.009G055900*, and *Potri.012G105500*) also showed a steady decline, but the decline continued during the entire 16 h auxin depletion period (Fig. 7B). Addition of 20 μM IAA to the growth medium induced gene expression within 30 min for all of the protease genes assayed. The levels of the induction varied among the genes, with *Potri.004G215400* showing a low level of induction while *Potri.012G105500* and *Potri.011G066900* showed the greatest level of induction. With the exception of *Potri.012G105500*, gene expression increased rapidly within 30 min and then began to decline.

### Bark N remobilization is associated with plastid glutamine synthetase expression

**Fig. 8.**
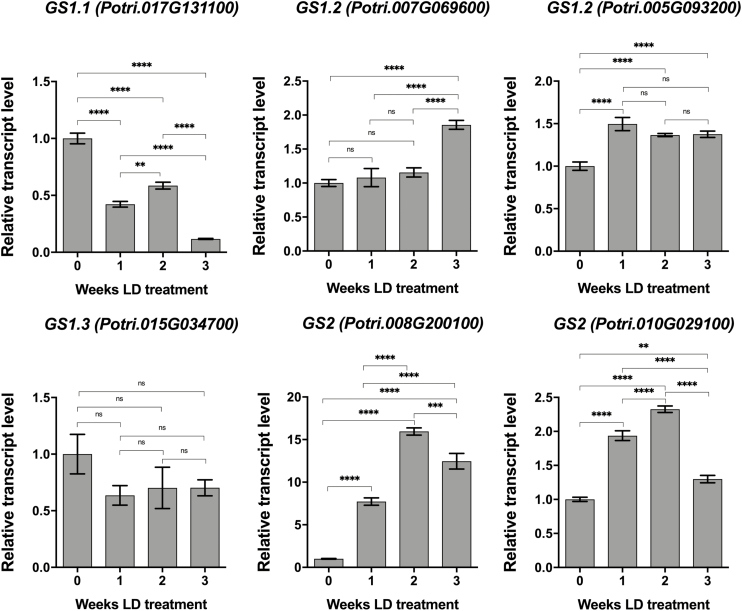
Glutamine synthetase (GS) expression in bark during N remobilization. Relative expression of genes encoding cytosolic (GS1) and plastid (GS2) forms of GS were determined by qRT-PCR at weekly time points during LD-mediated N remobilization. Plants (*Populus trichocarpa*, Nisqually) were first grown in LDs for ~6 weeks followed by SD treatment for 8 weeks at 20 °C to induce dormancy. Leaf senescence and abscission was then induced with 6 weeks of further SDs at low temperature (LT) (10 °C, light; 4 °C dark). After leaf senescence and abscission, the plants were placed in the dark at 4 °C for 7 weeks to release buds from dormancy. Renewed shoot growth was then initiated by placing plants in LDs at 20 °C. Three independent replicates were collected for RNA purification at the start of the LD treatment and at weekly intervals for 3 weeks. Error bars indicate the mean ±SE, and values significantly different from the 0 week LD treatment are indicated by *P<0.05; **P<0.01, ***P<0.001, and ****P<0.0001 based on ANOVA and Tukey’s multiple comparison test.

In poplar, glutamine is the primary amino acid transported during spring N remobilization (Sauter and van Cleve, 1992; Millard *et al.*, 2006), yet poplar BSP is composed of <2.5% glutamine. This suggests that BSP catabolism probably results in the synthesis of glutamine and other amino acids via the activity of glutamine synthetase (GS), NADH-glutamate synthase (GOGAT), and possibly glutamate dehydrogenase (GDH). In poplar, GS is encoded by a gene family of eight genes that include cytosolic (GS1) and chloroplastic (GS2) isoforms (Castro-Rodríguez *et al.*, 2011). The microarray data showed differences in expression of four genes encoding GS1 isoforms and two GS2 during N remobilization, while expression of the remaining two *GS1* genes (*Potri.004G085400* and *Potri.012G043900*) was not detected on the microarray. Little difference in the expression of GS1 isoforms during N remobilization was observed (Fig. 8), except for *GS1.1* (*Potri.017G131100*) which showed a gradual decline in expression during LD-mediated N remobilization. In contrast to *GS1* genes, both genes for the *GS2* isoforms (*Potri.008G200100* and *Potri.010G029100*) showed increased expression during N remobilization, with the greatest increase in expression occurring for *Potri.008g200100*. Thus, it appears that the synthesis of glutamine for N transport during N remobilization involves mostly plastid isoforms as opposed to cytosolic isoforms.

## Discussion

### BSP accumulation influences bud break and shoot growth following dormancy

Numerous studies have indirectly demonstrated the importance of internal N stores to meet the N demand of trees (Millard and Proe, 1991; Millard, 1994; Neilsen *et al.*, 1997; Weinbaum and Van Kessel, 1998; Guak *et al.*, 2003; Millard *et al.*, 2006). Utilization of internal N stores has the advantage of uncoupling growth from N uptake (Jing *et al.*, 2004; Cooke and Weih, 2005; Millard and Grelet, 2010), yet the relative contribution of different stores such as proteins and amino acids has yet to be established. In this research, a transgenic approach using RNAi-mediated gene knockdowns to reduce BSP bark accumulation showed that initial growth following dormancy was reduced when BSP accumulation was reduced. These results indicate that BSP is an important N store that is remobilized and used to support new shoot growth following dormancy. In addition to BSPs, poplars also accumulate amino acids, including arginine, in bark during overwintering (Wildhagen *et al.*, 2010). It is not known if RNAi-induced reductions in BSP accumulation via RNAi influenced amino acid accumulation during N storage but, based on the growth response of the RNAi plants, it appears that these other N storage pools are unable to compensate for reductions in BSP accumulation. Additional research is needed to understand the relationships between different N storage pools and whether compensatory mechanisms exist when specific N storage pools are altered.

Since BSP RNAi transgenic studies involved reduced BSP accumulation, which could potentially affect other N storage pools, an additional set of studies were conducted where the numbers of N sinks that compete for N during regrowth were altered while keeping N sources the same. Because initial spring shoot growth competes for N, then measuring growth parameters known to be influenced by N availability (i.e. leaf area and stem length) (Cooke *et al.*, 2005) may reveal growth differences resulting from N competition. However, this approach does not exclude the contribution that C reserves may make to shoot growth. Consistent with this hypothesis, when the number of growing shoots increased while N reserves remained constant, growth was reduced irrespective of shoot type (proleptic or sylleptic). Leaf area and sylleptic shoot development were most affected when shoot competition increased. Moreover, it appears that there is a differential response in the competition between shoot types since there was little, if any, competition from previously formed sylleptic shoots on shoots originating from axillary buds, while shoots originating from axillary buds competed with shoots from previously formed sylleptic shoots. Thus, it appears that shoots growing from axillary buds may be dominant to new shoots growing from previously formed sylleptic branches. These results also suggest that growth following dormancy is likely to be source limited as opposed to sink limited. If initial growth is source limited, then strategies to increase N storage capacity or efficiency could reduce source limitations to growth and improve productivity. Poplar sylleptic and proleptic shoots are known to differ in their physiology. Sylleptic shoots/branches export carbon (C) mainly to the lower stem and roots while the leaves on the main stem (proleptic) partition C that is mostly used for height growth (Scarascia-Mugnozza *et al.*, 1999). In addition, sylleptic shoots were reported to have a greater translocation efficiency than proleptic shoots (Scarascia-Mugnozza *et al.*, 1999). Our results suggest that besides differences in C partitioning, sylleptic and proleptic shoots also differ in their physiological responses related to tree N budgets.

Unexpectedly, the timing of bud break was delayed for the majority of transgenic poplars with reduced levels of BSPs, indicating that, in addition to shoot growth, N storage also impacts the timing of bud break. Poplar bud break involves the APETALA2/ERF gene *EBB1* (*EARLY BUD-BREAK1*) (Yordanov *et al.*, 2014). Interestingly, transcriptome analysis revealed that the most enriched GO category for putative targets for EBB1 were genes associated with N metabolism (Yordanov *et al.*, 2014). Thus, it appears that N metabolism, including remobilization from reserve stores, has an important role in the timing of bud break following dormancy. The influence of N storage on initial shoot growth combined with the effects on timing of bud break indicate that improved N storage or efficiency could enhance productivity by the combined effects on the timing of bud break as well as the initial growth of shoots.

### BSP accumulation also contributes to bark sink status during N partitioning from leaves

^13^N tracer experiments using BSP RNAi lines showed a reduction of ^13^N partitioning to stems during leaf senescence. This suggests that N sink strength was reduced in BSP RNAi plants and that BSP normally makes a significant contribution to maintaining N sink status in WT poplars. This fits with the fact that the stem N and protein tend to be high during winter dormancy, and the BSP RNAi line had less BSP for N storage in the lower stem. However, there was still substantial N partitioning to stems in BSP RNAi plants, suggesting that other mechanisms also contribute to N sink strength during dormancy induction. Additionally, it is possible that since BSP RNAi plants are not knockouts, a low level of BSP synthesis is occurring which contributes to this N partitioning. The fact that ^13^N partitioning to the lower stem did not increase under SD-LT conditions suggests that the stem may become a direct sink for exported leaf N as the stem matures, even while growth in younger parts of the stem is still under way. It was surprising that the total export of ^13^N from leaves to stems did not increase when the photoperiod was switched from LDs to SD-LT, as we had expected. The decline in leaf N content during SD-induced senescence without increased leaf N export suggests that a low rate of N export begins during leaf development and that N uptake into the leaf continues well beyond the point at which the leaf reaches maturity in order to balance export and maintain the level of N in the leaf. Then, during senescence, rather than up-regulating N loading into the phloem, the leaf ceases N uptake, and the decrease in N uptake is what causes leaf N to decline. Alternatively, the lack of apparent increase in N export from leaves could be due to isotopic dilution (Babst *et al.*, 2013) since the concentration of glutamine in poplar leaves increases during SD-induced senescence, especially in the veins (Couturier *et al.*, 2010).

### Polar auxin transport is required for N remobilization from bark during regrowth

We found that BSP catabolism and N remobilization during shoot regrowth were associated with induction of specific auxin transporter genes, *ABCB5*, *ABCB7*, *PIN4*, *PIN7*, *AUX5/LAX7*, and *AUX6/LAX3*. Besides auxin transporters, the Arabidopsis nitrate transporter NPF6.3 (AtNRT1.1) can also transport auxin in roots (Krouk *et al.*, 2010). Since specific members of the poplar NPF (NRT) gene family are induced during N remobilization, it is possible that they may also contribute to auxin transport, yet a role for auxin transport by NPF (NRT) transporters in organs other than roots is not well established. During active growth, young and expanding leaves serve as a major source of auxin (Sundberg and Uggla, 1998; Ljung *et al.*, 2001; Schrader *et al.*, 2003) and our results implicate these tissue as important sources of auxin that regulate BSP catabolism and N remobilization. Similar to our findings in bark, *PttLAX3*, the ortholog of *AUX5/LAX7* and *AUX6/LAX3*, has also been reported to be induced in wood-forming tissues of hybrid aspen (*P. tremula*×*tremuloides*) during regrowth in spring (Schrader *et al.*, 2003). Moreover, expression of both *AUX5/LAX7* and *AUX6/LAX3* was significantly reduced in plants lacking buds and in stems treated with NPA, and appears to be associated with reduced bark auxin levels and inhibition of BSP catabolism. It was also observed that *PIN4* and *PIN7*, which are orthologs to *PttPIN1* and *PttPIN2*, are induced in bark as growth resumed, and these genes have also been reported to be induced during wood formation in hybrid aspen (Schrader *et al.*, 2003). Similar to *AUX5/LAX7* and *AUX6/LAX3*, expression of both *PIN4* and *PIN7* was reduced in plants without buds and in stems treated with NPA. Expression of auxin transporter genes in wood-forming tissues is also known to be associated with increases in auxin transport capacity (Schrader *et al.*, 2003) and it seems likely that this relationship also occurs in bark. Thus, it appears that both wood-forming tissues and bark share components involved in auxin transport during regrowth in spring even though transported auxin influences different physiological and developmental processes in these two tissues. We also found that specific auxin biosynthesis genes in the *TAA/TAR* and *YUCCA* families were induced in the young and expanding leaves during regrowth. Removal of buds, which eliminated the sites of auxin production, significantly reduced expression of auxin transporter and response genes compared with intact plants. Although stem auxin is also radially transported in poplar stems and proposed to be synthesized by dividing cambial cells (Sundberg and Uggla, 1998; Schrader *et al.*, 2003; Spicer *et al.*, 2013), it appears that radially transported auxin in poplar stems does not play a major role in BSP catabolism and N remobilization in bark since we failed to observe BSP catabolism in bark below the site of NPA application. If radial transport from cambial cells had occurred and played a role in BSP catabolism, it would be expected that BSP levels would have declined in bark below the site of NPA treatment. Apparently, auxin transported to, and in, poplar stems has different roles that are related to positional or developmental cues.

### BSP catabolism involves induction of protease genes by auxin

BSP catabolism during regrowth involves the action of proteolytic enzymes in poplar bark. In order to identify proteases responsible for poplar BSP catabolism during regrowth, multidimensional protein identification technology (MudPIT) has been used to analyze bark protein changes during BSP catabolism (Islam *et al.*, 2015). Thirty proteases showed increased abundance in bark during BSP catabolism, including papain-like cysteine proteases, serine carboxypeptidases, and aspartyl proteases. Among those proteases, four of them showed increased transcript abundance in our DNA microarray, including two serine protease genes *Potri.002G120400* and *Potri.014G018900*, one cysteine protease gene *Potri.006G232900*, and one aspartyl protease gene *Potri.018G014800* (Islam *et al.*, 2015). However, a notable number of protease genes that showed induced gene expression by DNA microarray analysis were not detected in proteome analyses using MudPIT. There could be multiple reasons for these differences. First, the proteome analyses were limited to samples of two time points (LD for 1 week and 3 weeks), while the DNA microarray analysis involved bark samples of four time points. Secondly, the activation of some proteases is via post-translational processes (van der Hoorn, 2008) which would not involve changes in genes expression. As a possible example, the cysteine protease (*Potri.011G066900*) was detected in MudPIT proteome analysis in poplar bark during N remobilization (Islam *et al.*, 2015) yet the results of the current study indicate that expression of this gene changed little during N remobilization. Furthermore, expression of this cysteine protease gene was induced in plants lacking buds and was less affected by NPA treatment compared with the other protease genes that were studied. This suggests that for this specific protease gene, auxin has less of a role in regulating expression, although we cannot exclude the possibility that auxin may affect post-translational activation.

Specific proteases were found to be auxin inducible, further supporting the role of polar auxin transport in regulating BSP catabolism through auxin-mediated regulation of protease gene expression. For several of the protease genes, expression declined after the initial induction by IAA. This decline in expression was not detected for the *AUX/IAA* genes, suggesting differences in auxin-inducible expression between the protease and *AUX/IAA* genes. This difference may reflect the relative location in the signaling response pathway with the *AUX/IAA* genes in early steps of auxin signaling, while the protease genes are likely to be at or near the endpoint of the pathway. The results from the bud removal, NPA, and IAA treatments are consistent with a role for polar transport of auxin from shoots to bark in regulating BSP catabolism and N remobilization by regulating the expression of specific proteases.

### Glutamine synthesis and transport are involved in N remobilization

Previous studies have shown that the expression of GS genes is seasonally regulated in poplar (Castro-Rodríguez *et al.*, 2011). In our study, two GS2 genes *Potri.008G200100* and *Potri.010G029100* showed increased transcript levels in bark during regrowth. Changes in *GS2* expression have been previously reported in poplar bark during spring regrowth (Castro-Rodríguez *et al.*, 2011). Taken together, these results suggest that bark serves as an N source during regrowth and GS2 isoforms play an important role in N metabolism and remobilization, and in particular in the production of glutamine since it is the major amino acid transported during N remobilization. In addition, only a slight increase of *GS1.2* transcript levels was observed during regrowth, suggesting that *GS1.2* is not the major isoform involved in N remobilization. It has also been reported that expression of *GS1.1* is mostly associated with older leaves and increases during summer and autumn, indicating that GS1.1 may be involved in N metabolism and remobilization during leaf senescence (Castro-Rodríguez *et al.*, 2011). This may explain why the transcript levels of *GS1.1* were reduced in bark during regrowth. However, the transcript level of *GS1.3* was slightly reduced in bark during regrowth while it was increased during spring regrowth in a previous report (Castro-Rodríguez *et al.*, 2011), which may indicate that GS1.3 plays a role in N metabolism during active growth after N is remobilized from bark.

Glutamine is the major amino acid that is transported from bark to leaves during spring N remobilization (Sauter and van Cleve, 1992; Millard *et al.*, 2006; Wildhagen *et al.*, 2010). PtCAT11 has previously been shown to transport glutamine and seems to be involved in N remobilization from senescing leaves to bark during autumn (Couturier *et al.*, 2010). Our results revealed that *CAT11* expression was negatively related to N remobilization from poplar bark during regrowth. Moreover, we found that *CAT10* was induced in bark during regrowth and reduced when BSP catabolism was inhibited by bud removal or NPA treatment. The expression patterns of *CAT11* are consistent with a role in glutamine import to bark during leaf senescence, while *CAT10* may be involved in the export of glutamine during N remobilization from bark to shoots. Thus, CAT11 appears to be associated with bark sink status while CAT10 is related to bark source status.

**Fig. 9.**
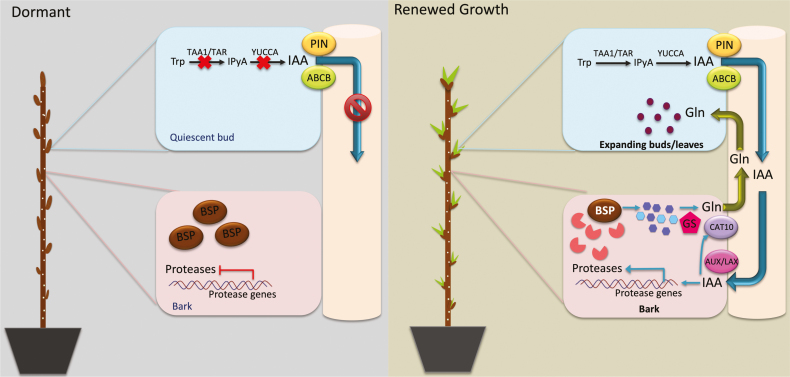
Possible model of auxin-regulated N remobilization during regrowth following dormancy in poplar. During dormancy, auxin biosynthesis is inhibited in dormant buds and shoots which in turn limits auxin transport to bark. The lack of auxin transport to bark blocks BSP catabolism and N remobilization. Once growth resumes following dormancy, auxin biosynthesis increases in expanding buds and shoots which serve as a source of auxin. Auxin is then transported from expanding buds and shoots to bark and activates the expression of protease genes and the production of proteases, resulting in BSP catabolism. The products of BSP catabolism serve as substrates for glutamine via activity of plastid-localized glutamine synthetase (GS). In addition transported auxin, or other metabolites, also appear to induce the expression of the *CAT10* amino acid transporter gene. Glutamine is then transported to expanding buds and shoots that serve as N sinks.

In summary, the results of this research indicate that BSP is central to N storage during dormancy, not only as a storage molecule, but also as a mechanism that contributes to sink strength in the autumn. Furthermore, when bark transitions from an N sink to an N source during release from dormancy, the catabolism of BSP contributes to the source strength of bark and can influence the timing of bud break. Our results also indicate that N remobilization from bark to shoots during the spring is regulated to a large extent by auxin. We propose a model for this process initiated by the production of auxin in expanding buds and shoots which moves by polar transport to bark (Fig. 9). Auxin then induces the expression of specific proteases that are involved in the catabolism of BSP. The products of this catabolism are used in the biosynthesis of glutamine which is then transported to expanding shoots which act as N sinks. This transport appears to involve CAT10, which has high sequence similarity to the demonstrated glutamine transporter CAT11. There are many unanswered questions about this process, but the results from this research provide a foundation for further research to define regulatory and transport factors involved in N remobilization in trees.

## Supplementary data

Supplementary data are available at *JXB* online.

Table S1. List of genes and primers used for qRT-PCR.

Table S2. GO enrichment table.

Table S3. Gene models and values of the Mapman hormone bin.

Fig. S1. Effect of NH_4_NO_4_ fertilization on growth and development of *Populus tremula*×*Populus alba* clone 717-1B4.

Fig. S2. Reduced BSP accumulation via RNAi knockdown reduces growth following bud break.

Fig. S3. Sink competition between axillary or sylleptic shoots affects poplar shoot growth following dormancy.

Fig. S4. Differential sink competition between axillary and sylleptic shoots.

Fig. S5. Growth and chlorophyll content of wild-type (WT) and BSP RNAi poplars.

Fig. S6. Global changes in bark gene expression during growth following dormancy.

Fig. S7. Clustered image maps of auxin-related genes.

Fig. S8. Clustered image maps of protease genes.

Fig. S9. Clustered image maps of amino acid transporter genes.

Fig. S10. Effect of different NPA concentration on bark BSP levels.

Fig. S11. Effect of different NPA concentrations on bark levels of IAA, IAA-alanine, NPA, and Trp.

eraa130_suppl_Supplementary_Figures_S1_S11

eraa130_suppl_Supplementary_Table_S1

eraa130_suppl_Supplementary_Table_S2

eraa130_suppl_Supplementary_Table_S3

## Data Availability

All data and materials will be made available to researchers upon request.
