# Supplementary material for: Seasonal nitrogen remobilization and the role of auxin transport in poplar trees
Source: J Exp Bot. 2020 Mar 12;71(15):4512–30. doi: 10.1093/jxb/eraa130 (PMC7382381; doi:10.1093/jxb/eraa130)
Supplement: eraa130_suppl_Supplementary_Figures_S1_S11 [file eraa130_suppl_supplementary_figures_s1_s11.pdf]

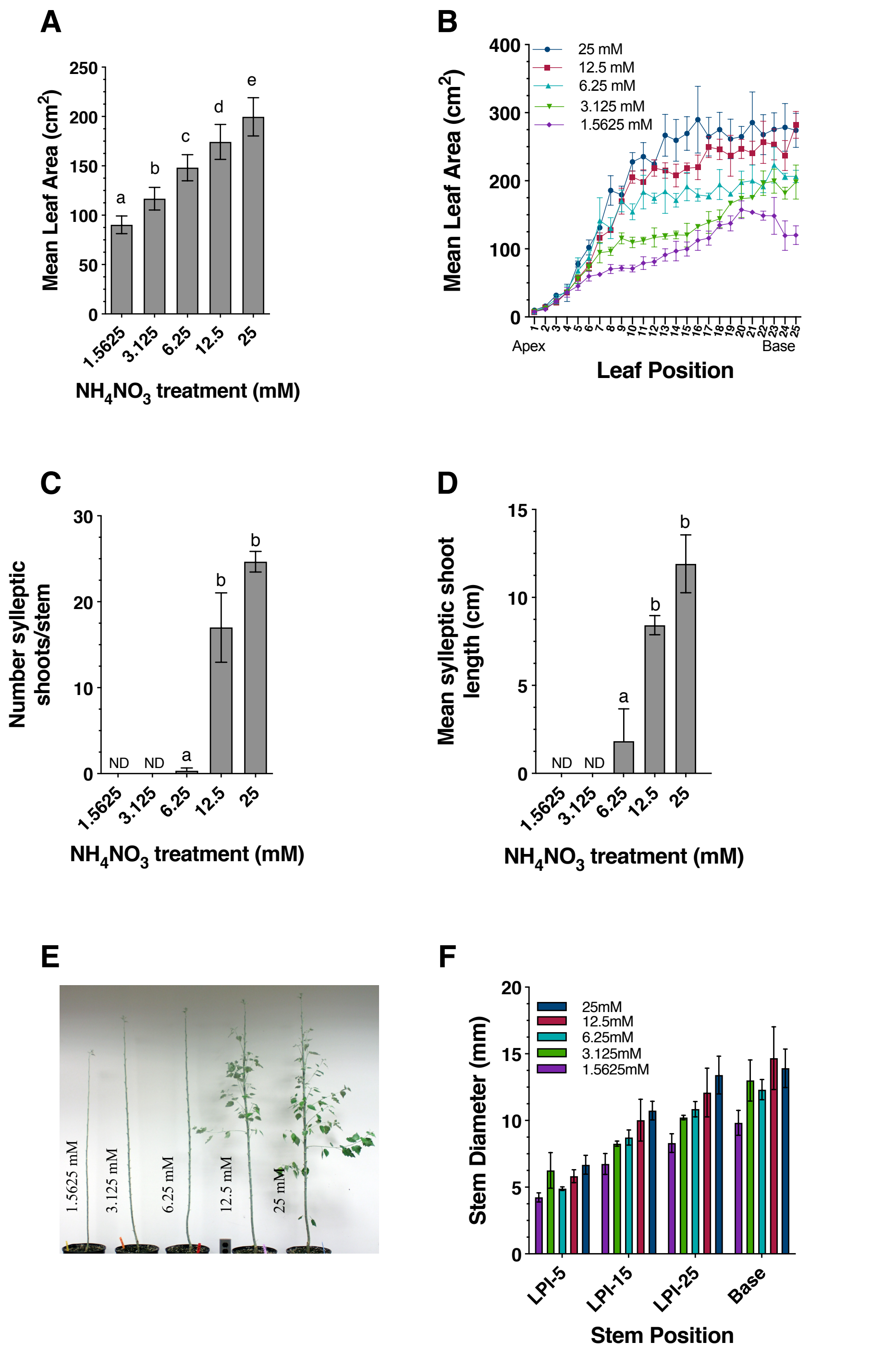

Fig. S1. Effect of NH_4NO_3 fertilization on growth and development of *Populus tremula x Populus alba* clone 717-1B4. (A) Mean leaf area per plant; (B) mean leaf area by leaf position (apex to base); (C) mean number of sylleptic shoots per stem; (D) mean length of sylleptic shoots per stem; (E) examples of sylleptic shoot growth (proleptic shoot leaves removed for clarity); and (F) stem diameter at different stem positions and at different NH_4NO_3 fertilization rates. Error bars indicate mean SE and letters indicate values significantly different based on ANOVA and Tukey's multiple comparison test at $p<0.01$, ND specifies not detected.

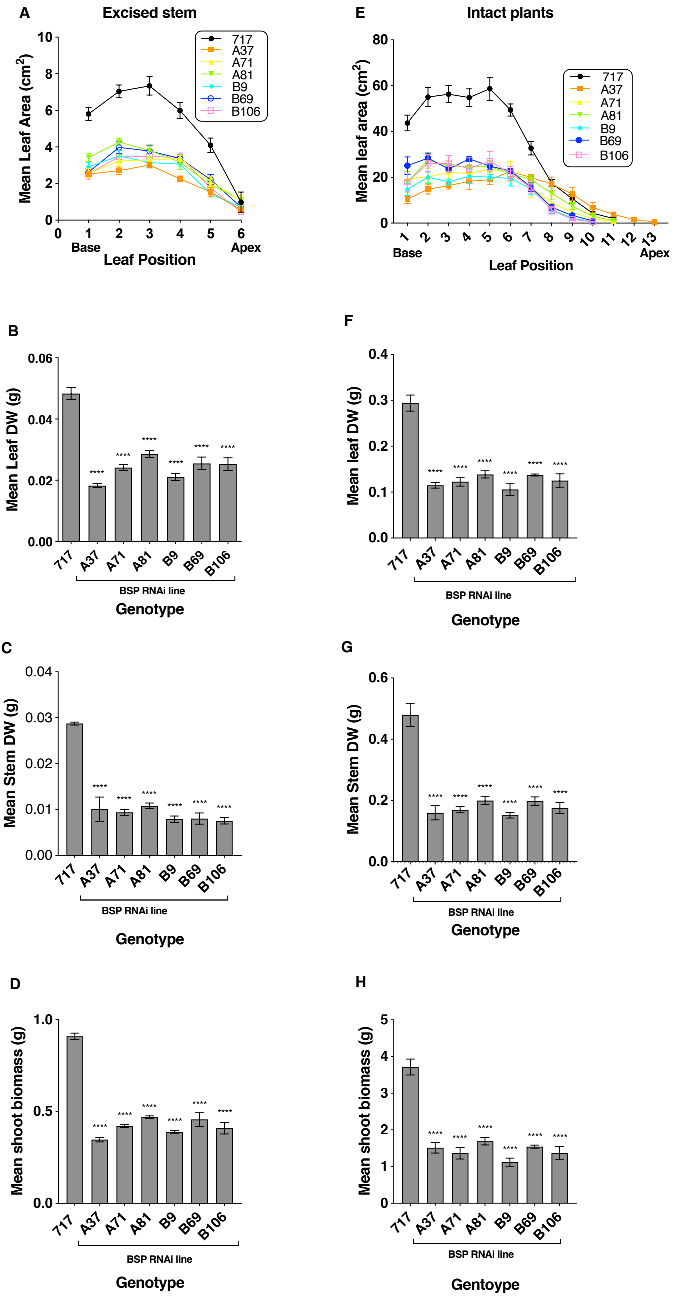

Fig. S2. Reduced BSP accumulation via RNAi knockdown reduces growth following bud break. (A) Mean leaf area at different leaf positions; (B) mean leaf dry weight (DW); (C) mean stem DW and (D) mean shoot biomass of new growth 6 weeks after bud break of excised stems for control (717) and BSP-RNAi lines (A37, A71, A81, B9, B69, B106). (E) Mean leaf area at different leaf positions; (F) mean leaf DW; (G) mean stem DW and (H) mean shoot biomass of new stem growth 6 weeks after bud break for intact plants. Error bars indicate mean SE and **** indicates means significantly different from control (717) at $p<0.0001$ based on ANOVA and Tukey's multiple comparison tests.

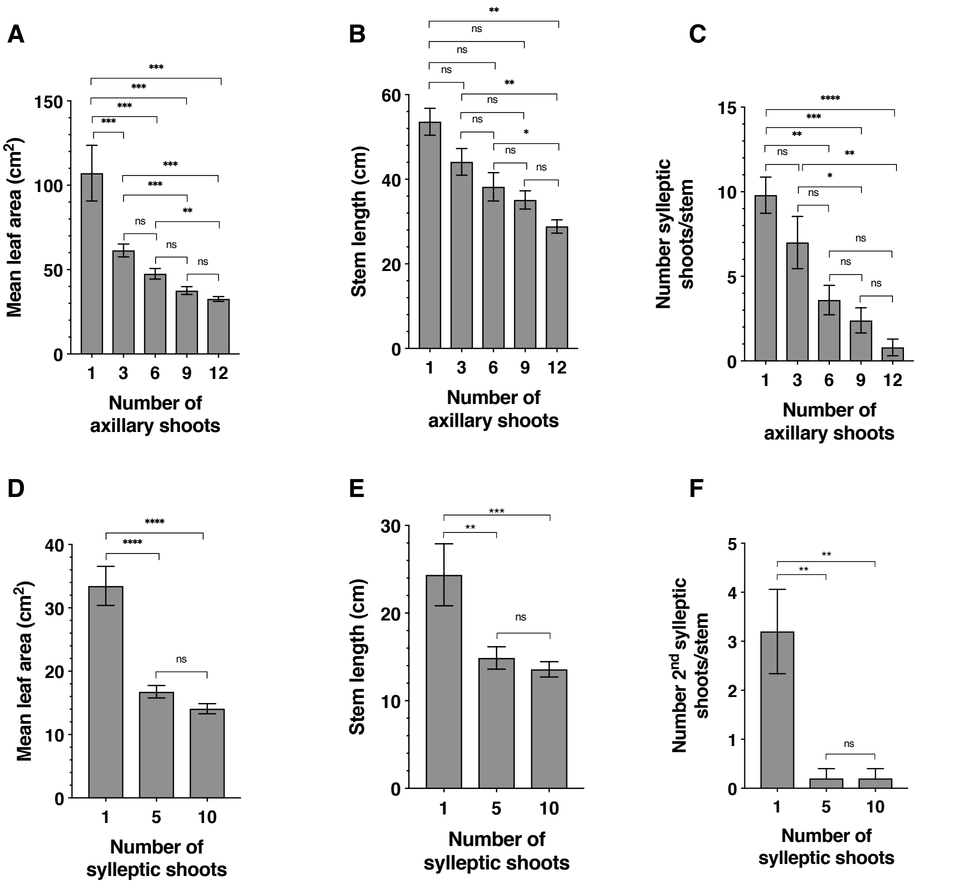

Fig. S3. Sink competition between axillary or sylleptic shoots affects poplar shoot growth following dormancy. Shoot N-sinks were manipulated by removal of either axillary buds or sylleptic shoots prior to regrowth following dormancy of *Populus trichocarpa* (Nisqually) plants. 6 weeks after bud break (A) mean leaf area of shoots from axillary buds; (B) stem length of shoots from axillary buds; (C) number of sylleptic shoots per stem of shoots from axillary buds; (D) mean leaf area of shoots from previous sylleptic shoots; (E) stem length of shoots from previous sylleptic shoots; and (F) number of secondary sylleptic shoots growing from previous sylleptic shoots was measured. Error bars indicate mean SE and significant differences between treatments are indicated by * $p<0.05$, ** $p<0.01$, *** $p<0.001$, **** $p<0.0001$ based on ANOVA and Tukey's multiple comparison test, ns indicates no significant differences.

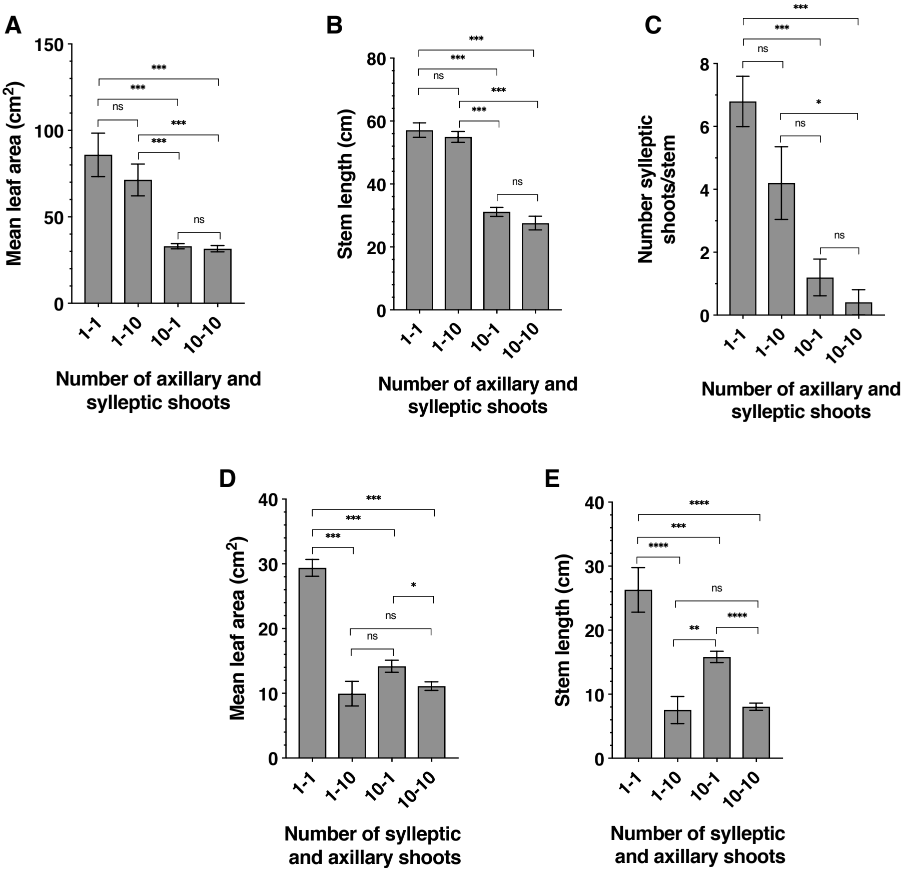

Fig. S4. Differential sink competition between axillary and sylleptic shoots. Shoot sinks were manipulated by the removal of either axillary buds or sylleptic shoots prior to regrowth following dormancy of *Populus trichocarpa* (Nisqually) plants. 6 weeks after bud break (A) mean leaf area of shoots from axillary buds of plants with either 1 axillary bud and 1 sylleptic shoot (1-1), 1 axillary bud and 10 sylleptic shoots (1-10), 10 axillary buds and 1 sylleptic shoot (10-1) or 10 axillary buds and 10 sylleptic shoots (10-10); (B) stem length of shoots from axillary buds of plants with either 1 or 10 axillary and sylleptic shoots as described in A; (C) number of sylleptic shoots per stem of shoots from axillary buds of plants with either 1 or 10 axillary and sylleptic shoots as described in A; (D) mean leaf area of shoots from previously formed sylleptic shoots of plants with either 1 sylleptic shoot and 1 axillary bud (1-1), 1 sylleptic shoot and 10 axillary buds (1-10), 10 sylleptic shoots and 1 axillary bud (10-1), or 10 sylleptic shoots and 10 axillary buds (10-10); (E) stem length of shoots from previously formed sylleptic shoots of plants with either 1 or 10 sylleptic shoots and axillary buds as described in D. Error bars indicate mean SE and significant differences between treatments are indicated by * $p<0.05$, ** $p<0.01$, *** $p<0.001$, **** $p<0.0001$ based on ANOVA and Tukey's multiple comparison test, ns indicates no significant differences.

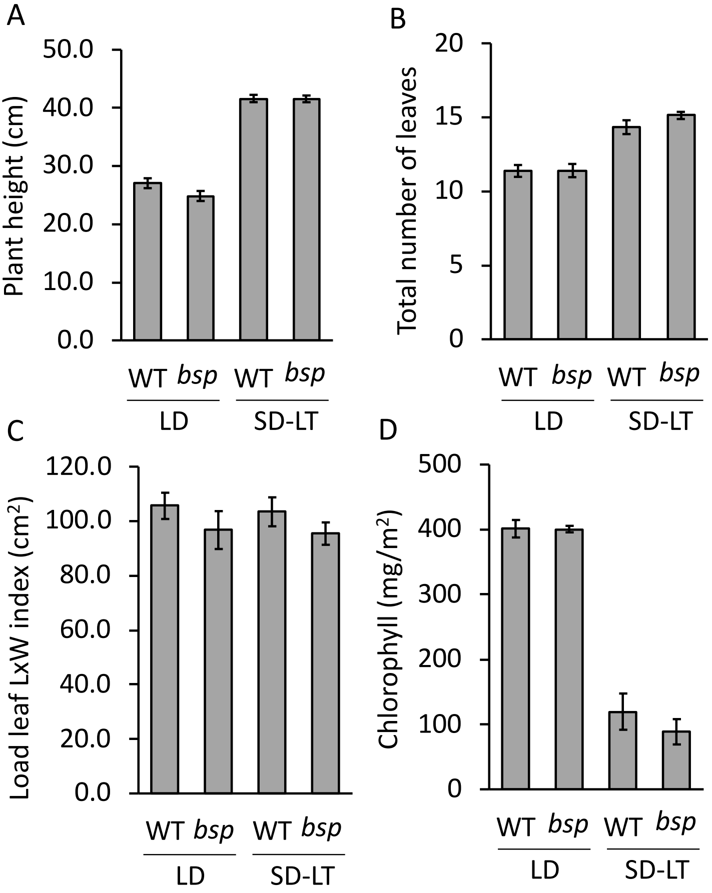

Fig. S5. Growth and chlorophyll content of wild-type (WT) and BSP RNAi (*bsp*) poplars. Plants were grown in long-day photoperiods (LD), or grown first in LD followed by 6 weeks of short-day (SD) photoperiod plus another 3-5 weeks in SD and low temperature (SD-LT) and (A) plant height; (B) leaf number; (C) load leaf LxW index; and (D) load leaf leaf chlorophyll content were measured before the ^{13}N experiments and the load leaf was the leaf that was fed $^{13}NH_3$. Bars indicate means SE.

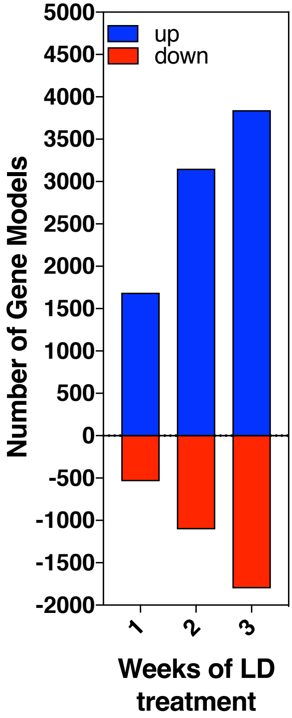

Fig. S6. Global changes in bark gene expression during growth following dormancy. Number of genes either up-regulated or down-regulated in the bark of *Populus trichocarpa* (Nisqually) after either 1, 2, or 3 weeks of LD treatment following dormancy. Plants were first treated with SD and SD combined with LT to induced dormancy followed by LT to overcome dormancy (see materials and methods) prior to LD treatment. Gene expression was compared to that in the bark immediately prior to the LD treatment of plants that had receive sufficient LT treatment to overcome dormancy. Both up-regulated and down-regulated genes were determined based on a *P*-value 0.01 and a log2-fold change ≥ 1.585.

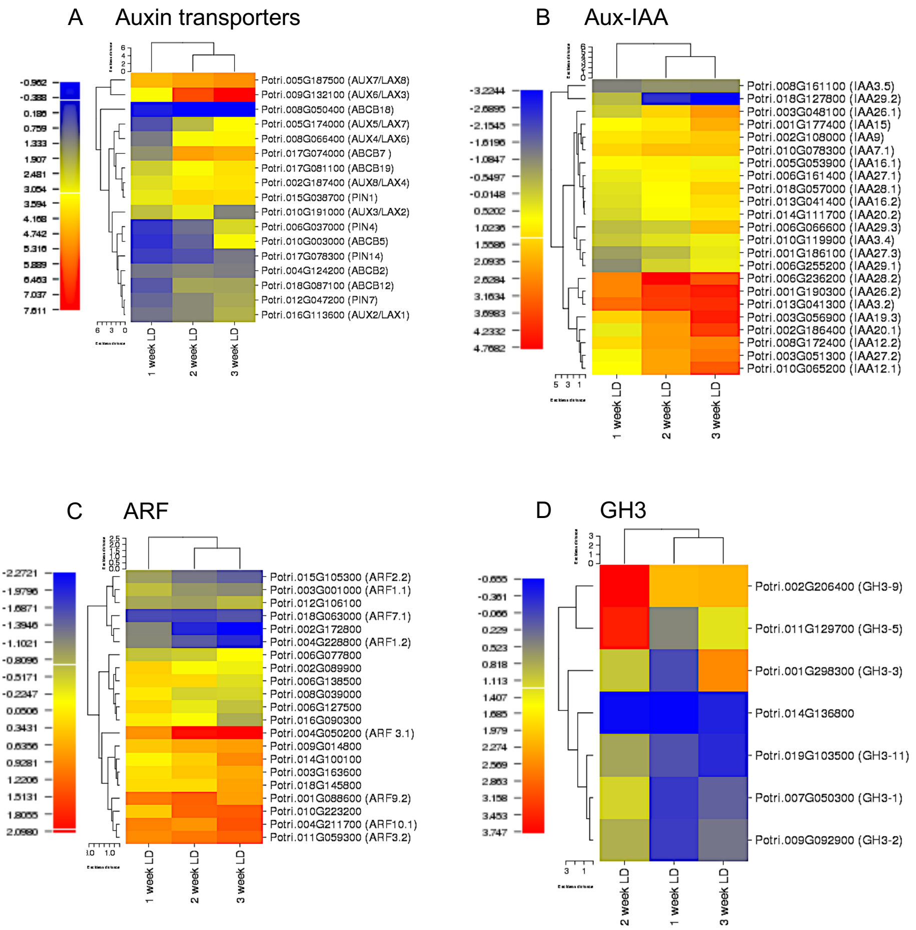

Fig. S7. Clustered image maps of auxin transporter (**A**), Aux-IAA (**B**), ARF (**C**) and GH3 (**D**) genes expressed in poplar bark after 1, 2 and 3 weeks of LD-mediated regrowth following dormancy. Gene expression (log2-fold and P-value ≤0.05) were compared to that in the bark immediately prior to the LD treatment of plants that had received sufficient LT treatment to overcome dormancy.

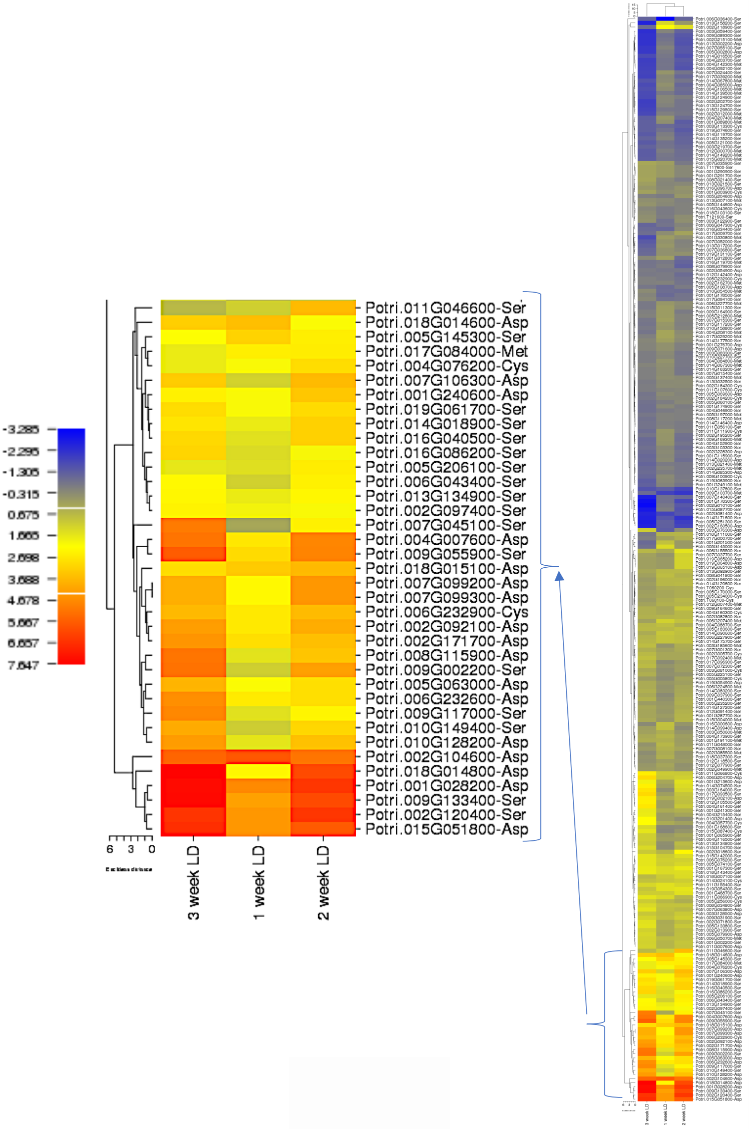

Fig. S8. Clustered image maps of protease genes expressed in poplar bark after 1, 2 and 3 weeks of LD-mediated regrowth following dormancy. Gene expression (log2-fold and P-value ≤0.05) was compared to that in the bark immediately prior to the LD treatment of plants that had received sufficient LT treatment to overcome dormancy.

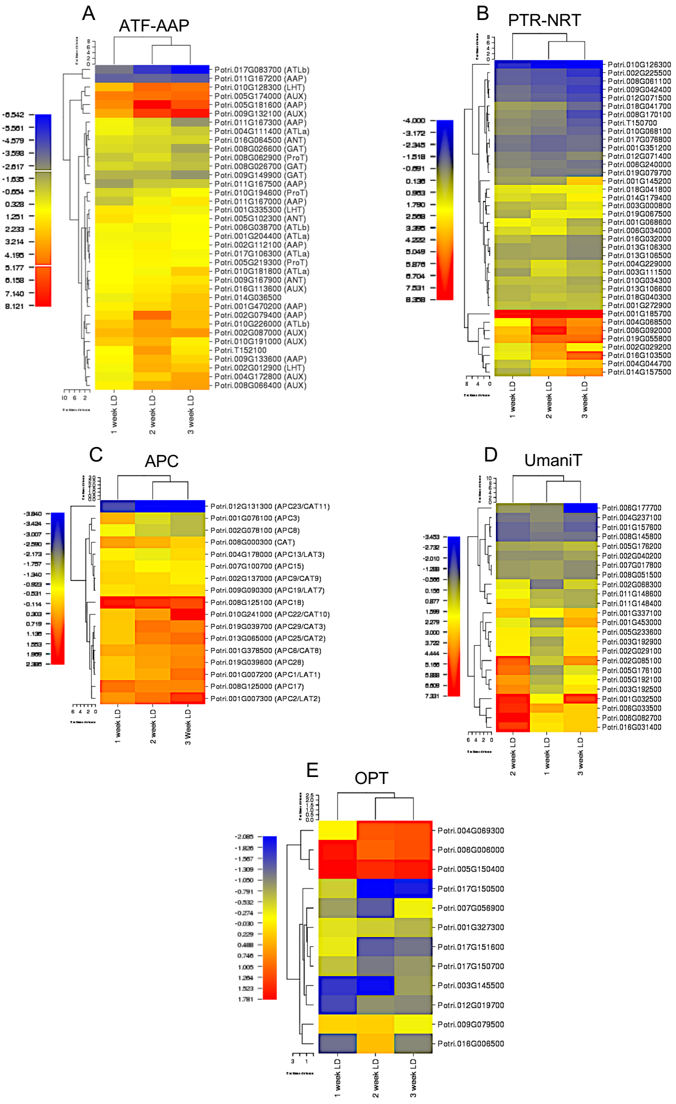

Fig. S9. Clustered image maps of amino acid transporter genes in the (**A**) ATF-AAP, (**B**), PTR-NRT (**C**) APC, (**D**) UmaniT, and (**D**) OPT families expressed in poplar bark after 1, 2 and 3 weeks of LD-mediated regrowth following dormancy. Gene expression (log2-fold and P-value ≤0.05) was compared to that in the bark immediately prior to the LD treatment of plants that had received sufficient LT treatment to overcome dormancy.

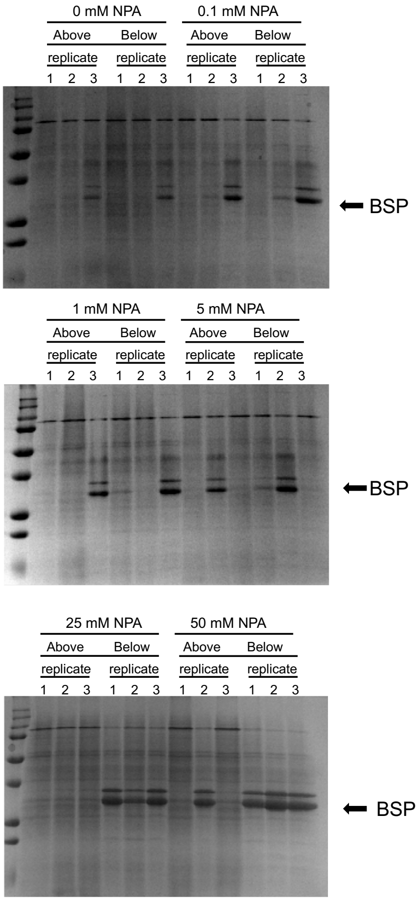

Fig. S10. Effect of different NPA concentrations on the abundance of BSP in poplar bark during LD N remobilization. Plants (*Populus trichocarpa*, Nisqually) were first grown in LD for approximately 6 weeks followed by SD treatment for 8 weeks at 20C to induce dormancy. Leaf senescence and abscission was then induced with 6 weeks of further SD at low-temperature (LT) (10C, light; 4C dark). After leaf senescence and abscission the plants were place in the dark at 4C for 7 weeks to release buds from dormancy. Renewed shoot growth was then initiated by placing plants in LD at 20C. Stems of plants were then ringed with 0, 0.1, 1.0, 5.0, 25, or 50 mM NPA when placed in LD to initiate new shoot growth. After 3 weeks of LD treatment bark was collected and soluble bark proteins were assayed by SDS-PAGE.

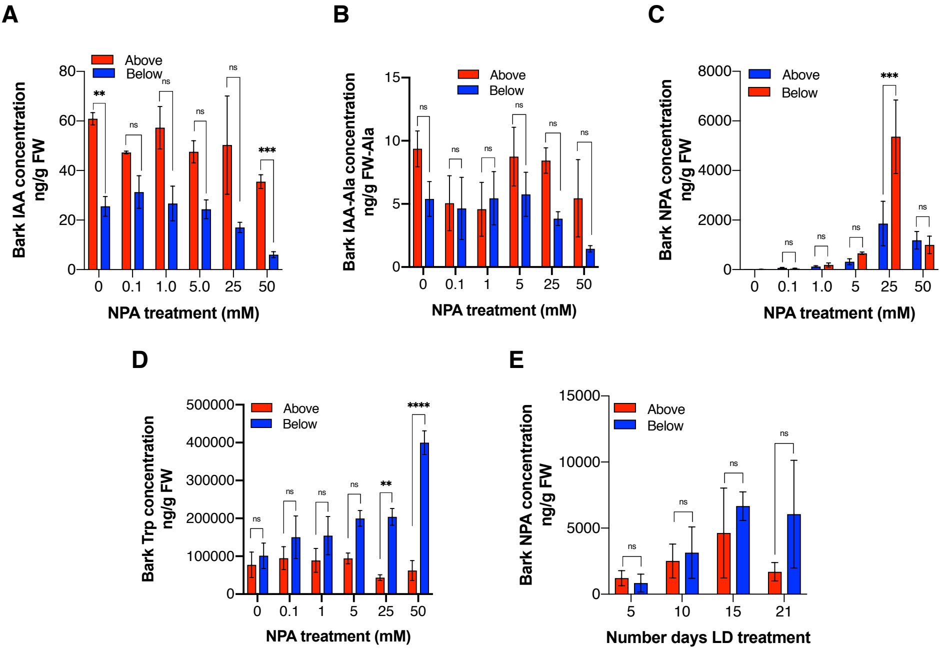

Fig. S11. Effect of different NPA concentrations on the abundance of bark (**A**) IAA, (**B**) IAA-Alanine, (**C**) NPA, (**D**) Trp, above and below the site of treatment, and (**E**) bark NPA content above and below the site of treatment after 5, 10, 15 or 21 days of NPA treatment. Metabolites and NPA levels were measured by LC-MS/MS after 21 days of LD-treatment following dormancy release except for **E** which was measured at the time interval indicted. Error bars indicate mean SE; ND indicates below detection limits; values significantly different between bark above or below the site of NPA treatment are indicated by * p<0.05; ** p<0.01, *** p<0.001, and p<0.0001 based on multiple t-tests and Holm-Sidak multiple comparison test.
