# Supplementary material for: Seasonal nitrogen remobilization and the role of auxin transport in poplar trees
Source: J Exp Bot. 2020 Mar 12;71(15):4512–30. doi: 10.1093/jxb/eraa130 (PMC7382381; doi:10.1093/jxb/eraa130)
Supplement: eraa130_suppl_Supplementary_Table_S1 [file eraa130_suppl_supplementary_table_s1.pdf]

Supplemental Table 1. Primers used for qRT-PCR

Gene name	Gene model	Forward primer	Reverse primer
CDC2	Potri.004G133500	ATTCCCCAAGTGGCCTTCTAAG	TATTCATGCTCCAAAGCACTCC
CYC063	Potri.005G240200	CCTGGCACTAATGGGTCTCAG	CACAACTCTTCCGAACACCAC
PT1	Potri.002G127700	GCGGAAAGAAAAACTGCAAG	TGACAGCACAGCCCAATAAG
TIP4-like	Potri.009G093200	GCTGATAATGGGGTGTCG	CAACTCTAAGCCAGAATCGC
ABCB5	Potri.010G003000	GGAAAGGAAGGAATCGCAACGG	CGGTCACCTACAACTGTGTCATAAC
ABCB7	Potri.017G074000	GCAATAGCCCGTGCCATACTG	GCATCTTGAACAACCTTCTCCGAC
AUX5/LAX7	Potri.004G172800	GCAGTGGAGCAGCCATCAAAG	CGAACCCAACAATCAGCACCC
AUX6/LAX3	Potri.009G132100	GCCTCGTTATGACTACTTACACTGC	AGCACTATTTTGGTTGGACCCG
PIN4	Potri.005G187500	GCGTCGTCTGATGCTTACTCAC	CGGACCCAAAAAGGCGTGTTC
PIN7	Potri.012G047200	GATGTTAGGCTCGCTGTATCTCC	AGCACCAACTTTCTCACCTTCAG
IAA12.1	Potri.010G065200	GATGGTGCTCCTTATCTCAG	CCTTCCCTCTCCGAATACTC
IAA19.3	Potri.003G056900	GCAGCGATGATGGACATAAAAATG	CCCCACAACTTGACTCTTTG
IAA20.1	Potri.002G186400	GGGGAAGGCAGTAACATCAGAAG	CCATCGTGGGCTAATAGGTTCAG
IAA26.2	Potri.001G190300	ACTCACAAGTTTCCATCTTCC	CTACCACCGCTTTAGTGC
IAA29.2	Potri.018G127800	TAGTCGTCAAAGCAGCAGGGCG	GTTGGAAAAGAATGGTGGAGGC
TAA/TAR1	Potri.010G044500	CACCCCGTCAGTCTTGTAGC	GCACCATCAGGATTATTTGGCG
TAA/TAR1	Potri.008G187800	CGTGGCTGCTGCTCCTTATTAC	TTCCCTTCCCCGAGGTTCAC
TAR2	Potri.015G081900	CAGCGGAACCCCTTAGTGTTG	GCCTGACAAACCCATCTGGATTG
TAR2	Potri.012G083300	CCTCCACAGTTGATAGCGATGG	AAGAACCAGCAAAGGCTCCC
YUCCA1	Potri.006G248200	CAACCAAGCCGTAAAAACGACAG	TCTCGCCAGTAGCAACAATAAGC
YUCCA2	Potri.006G243400	CGCCTTCACTTACCGAAAC	CGTGGTCTAATCTCAAACTTGC
YUCCA3	Potri.008G174600	TGACTTCTTTGCTCGGCGATG	ATGGCACCCCTTGTCCTCTAAG
YUCCA4	Potri.018G033200	TGAACTCCCGCTTCTTGGTTTC	CTGCCTTTTTTACAGCCTGGTTG
YUCCA5	Potri.007G028200	TTCCCTCCTGAAGTCCCAGC	TCTCCAGTTGCTACGATAAACCAC
YUCCA6	Potri.018G036800	AGGAGACCGAGTATGTGTGC	ACTTGTATGCTTTATGTCCCCTC
YUCCA7	Potri.010G062400	GGACTTCCCATTTCCAGAGGAG	GCCACAAACCAAAGGTCTCATC
YUCCA8	Potri.002G254200	CAAAAACGCACCTACGACAGG	CCTTGCTGACTGAACATACTCATTG
YUCCA9	Potri.005G186100	ACTCCAGCCAGTATGCGAATG	GGTATGAGCACCCCAATAGAGC
YUCCA10	Potri.005G111800	CAACCGTCTCTCCATCCCTAAC	GCAAGTAATCAATGAAACCCCTCC
YUCCA11	Potri.016G003300	AGAAAGGGAGGGTTGTTGTGG	TGTTGGTGCGTTAGAAGGGTAG
YUCCA12	Potri.002G207400	GCTTACGGGAAGTATCCAGTTTTCG	ACAATGGTGTCAAAGGGGTGTG
Protease-Ser	Potri.004G215400	AGGTGGGTAAATGGCTTGGC	AGAGGACCGTGACTTTTCAGC
Protease-Ser	Potri.009G002200	GTTACACCTCGTTTTCATTGGGTC	GGTCTTGACAGCAACAACAGTG
Protease-Ser	Potri.009G055900	GAAGCAGCAGGCAAGGATAC	TTTGGTGACATTGGCGTGC
Protease-Ser	Potri.012G105500	CAGTCCTCTCACAATCAAAGG	GCACCTCATAATCCAGAATGTTGC
Protease-Ser	Potri.010G149400	TCTCTGTCCTCCCAATCTACAC	TGAAACCAAGAACGCCAGG
Protease-Cys	Potri.011G066900	TATGACGGTGACTGCTCTGGTG	AATAACCTTCCATTCCCCACTCC
CAT10	Potri.010G241000	AGCAGACCCAAAGAACCCATC	TGAGGCAATAGAGAACGGTGAC
CAT11	Potri.012G131300	ACAAGAAGATTGATGAGGATGCCC	GCAGAAACGAGCAGAACTGTTG

Table 1. Primers used for qRT-PCR (Continued)

Gene name	Gene model	Forward primer	Reverse primer
GH3-3	Potri.001G298300	TGAGTTGGGTAAGGAATACGAGC	GAGGGGCTGAGTTGTGGAAC
GH3-5	Potri.011G129700	AGCACCCATTTCAAGGACAGG	GCTGGCAAAGACCACAAAGC
GH3-6	Potri.001G410400	GATGAGGTCCAAAAGAAGGTTC	AGGTAATCACAGGCACGG
GH3-9	Potri.002G206400	GTCTTGATGAGGGCAAAGCAATG	ACGGGTTCGGCACTTGAAG
GS1.1	Potri.017G131100	TGTCATTTGCGATACTTACACTCC	AGCACCACAGTAGTAAGGTCC
GS1.2	Potri.007G069600	GGGGAAGACAGTGAGGTCATTTTG	TCAGCAGCAATAGCAGGGTTG
GS1.2	Potri.005G093200	TAGCGACCCTGTTGTTGCC	CGAAGGATTTGTCAGCCCCTAC
GS1.3	Potri.015G034700	CCAACTAACAAGAGACACAGCG	CAGCACCAGCACCACAATAG
GS2	Potri.008G200100	TGTTAGCGGCACCAATGGG	GAGTGAGAGCACGACACCAG
GS2	Potri.010G029100	TGGTGTTGTGCTCTCACTCG	TTGCTTCAAACCCTCCTTCCTC
